# The perceptual saliency of fearful eyes and smiles: A signal detection study

**DOI:** 10.1371/journal.pone.0173199

**Published:** 2017-03-07

**Authors:** Mahmoud Medhat Elsherif, Muhammet Ikbal Saban, Pia Rotshtein

**Affiliations:** 1 School of Psychology, University of Birmingham, Birmingham, United Kingdom; 2 Department of Experimental Psychology, Ghent University, Ghent, Belgium; University of Akron, UNITED STATES

## Abstract

Facial features differ in the amount of expressive information they convey. Specifically, eyes are argued to be essential for fear recognition, while smiles are crucial for recognising happy expressions. In three experiments, we tested whether expression modulates the perceptual saliency of diagnostic facial features and whether the feature’s saliency depends on the face configuration. Participants were presented with masked facial features or noise at perceptual conscious threshold. The task was to indicate whether eyes (experiments 1-3A) or a mouth (experiment 3B) was present. The expression of the face and its configuration (i.e. spatial arrangement of the features) were manipulated. Experiment 1 compared fearful with neutral expressions, experiments 2 and 3 compared fearful versus happy expressions. The detection accuracy data was analysed using Signal Detection Theory (SDT), to examine the effects of expression and configuration on perceptual precision (d’) and response bias (c), separately. Across all three experiments, fearful eyes were detected better (higher d’) than neutral and happy eyes. Eyes were more precisely detected than mouths, whereas smiles were detected better than fearful mouths. The configuration of the features had no consistent effects across the experiments on the ability to detect expressive features. But facial configuration affected consistently the response bias. Participants used a more liberal criterion for detecting the eyes in canonical configuration and fearful expression. Finally, the power in low spatial frequency of a feature predicted its discriminability index. The results suggest that expressive features are perceptually more salient with a higher d’ due to changes at the low-level visual properties, with emotions and configuration affecting perception through top-down processes, as reflected by the response bias.

## Introduction

Facial expressions allow humans to extrapolate cues for navigating the social world. It enables people to infer mental states of others and, in turn, adjust their behaviour to match another’s emotional state. It is suggested that different facial features play a prominent role in the recognition of emotion [1, 2]. For example, the eyes are perceived as a diagnostic region for fear, whereas a smile reflects happiness [2, 3]. An inability to spontaneously fixate on the eyes impairs the ability to recognize fearful expression as seen in autism [3] and schizophrenia [4]. Similarly, an inability to spontaneously fixate on the mouth reduces recognition of happy expressions [5]. However, the underlying reason for spontaneous fixation on these diagnostic features is unclear. Two explanations have been proposed to account for the saliency of expressive features: affective- and visual-based hypotheses [6]. The current paper aimed to test the second hypothesis, namely the notion that different facial features have different perceptual saliency which is emphasized by the expressions. Specifically, we focused on the ability to detect fearful eyes and smiles presented around the conscious threshold.

The affective-based hypothesis assumes that the emotional content of the face explains the different patterns of expression processing. For example, Öhman and Mineka [7] stated that humans are biologically “hard-wired” to recognize fearful facial expression as it signals threat and the early detection of this threat signal can be crucial for survival [8]. Similarly, Kraut and Johnston [9] suggested that smiles serve as a social communicative tool signalling non-hostile intention, promoting friendliness facilitating social affiliation. Together the affective-based accounts suggest that expressive features derive their perceptual saliency from their relevance, as they convey an emotional meaning and diagnostic properties.

In contrast to the affective-based hypothesis, the visual-based hypothesis postulates that the early prioritization of expressive features is not dependent on the processing of the emotional meaning of the stimuli, but results from their low-level physical characteristics–bottom-up based saliency [6]. This account proposes that the visual properties of expressive features such as their visual contrast, provide a processing advantage. High contrast stimuli are perceptually salient (e.g., [10]). The perception of contrast is primarily mediated via low spatial frequencies (LSF) projected through the magnocellular pathway [11]. A stronger power of LSF reflect a higher energy stimulus with higher contrast leading to stronger activation in the visual cortex [12]. An increase in contrast (e.g., LSF power) of an expressive feature, can, therefore lead to its processing prioritisation. For example, the visual properties of the eyes and especially fearful eyes are considered salient [13]. The structure of the human eye has relatively large exposed white sclera with a horizontal elongation that is contrasted with a darkened coloured iris [14]. The contrast between the iris and sclera is magnified in a fearful expression. This could explain why eyes are so perceptually salient and especially fearful eyes. Similarly, humans and other primates often bare their teeth when they smile, enhancing the contrast between the white teeth and the darker lips. This may lead to perceptual prioritization of smiling mouth.

Two predictions can be derived from the visual-based account: first, the detectability of diagnostically expressive features should be higher and more precise than non-expressive features. Critically, this should be observed in a situation where the affective information conveyed by the feature/face is irrelevant to the task. Second, the detectability of expressive features should be independent of the facial configuration context. In other words, the prioritization of expressive features should depend on their basic-visual level properties, irrespective of their spatial location and their relation to other features. Thus, detecting an expressive feature would be similar in the context of a canonical (e.g. eyes above nose above mouth) and non-canonical face configuration. The aim of the current study was to test the above predictions of the visual-based hypothesis for eyes and mouth; which are considered diagnostic for fearful and happy affective states, respectively [2]. We next briefly review previous literature related to the processing of expression and specifically expressive eyes and mouth.

### Prioritization of emotional stimuli

It is debated whether processing of emotional information depends on the availability of cognitive resources, in other words is it automatic [15–17]. We note that the affective-based account for facial expression advocates automaticity [6–8]. Similarly, a visual-based account argues that the bottom-up physical property of stimuli captures attention (e.g. pop-out search). Thus, automaticity is not a unique prediction for the visual-based account. However, it remains an open question whether expressive features have developed in way that enhances their physical property to ensure they capture attention (like a screaming sound). The debate on automaticity can be re-phrased as the type of information contributing to the saliency maps of the environment. Saliency maps are ‘used’ by the brain to prioritise sensory processing. These maps are the product of the interplay between bottom-up and top-down processing. Bottom-up processes echoes the stimulus physical properties, while top down reflects the viewer’s input in relation to the stimulus’s physical properties [18]. In the context of the current study, if emotional values reflect the relevance of the stimulus, they should influence top-down processes, while the visual properties should influence bottom-up mechanisms.

### Prioritization of eyes and fearful eyes

The eyes are important in processing faces. Healthy observers are inclined to fixate primarily on the eyes, when viewing static faces (e.g. [4, 19]) and dynamic faces, (e.g., 20]), and during real-life interactions (e.g. [21, 22]). This is already evident in infants, who spontaneously fixate on the eyes [23]. Prioritizing information from the eyes appears to have evolutionary roots as similar gaze pattern has been reported in rhesus monkeys [24, 25] and orangutans [26]. It is argued that the eyes are important as they provide a window to the mind, the mental and emotional state of another (see review by [27], but see [6], whom suggest the mouth is diagnostically more informative).

Smith et al. [2] used the ‘Bubbles’ technique to decode the diagnostic features of the six basic facial expressions [28]. Bubbles [29] are masks of various spatial frequencies which are overlaid randomly on an image to occlude parts of the face. Participants are asked to recognize the expression presented in these partially masked images. Averaging across images that are correctly recognized reveals the diagnostic features that are essential for the recognition of a specific expression (e.g., mouth for smiles and eyes for fear). Using this method, the authors [2] showed that the eyes are fundamental to the recognition of fear. Similarly, it is shown that recognising fear is easier based on information from the top (where the eye are located) rather than from the bottom part of the face [30].

Eye-tracking experiments have been used to demonstrate the importance of the eyes when attempting to recognize a facial expression [31–34]. For instance, Gillespie et al. [32] reported higher dwell time on fearful eyes relative to most other expressions, including happy and neutral eyes. There was also a larger dwell time difference of eyes minus mouth for fearful relative to happy expressions [31]. Moreover, Schurgin and colleagues [33] showed that the task context rather than the stimuli affected the fixation pattern. Participants fixated for equal durations on fearful and neutral eyes when they had to decide whether a given expression was fearful or neutral. Taken together, the above studies demonstrate that when participants aimed to recognise expressions, information from the eyes support the recognition of fear. The aforementioned studies asked participants to explicitly recognise the facial expression. In these cases, the affective content of the face is a relevant dimension for the participants. Thus, it may not be surprising that eyes are perceived as a diagnostic feature of fear, as it receives processing priority in this recognition context.

Within this framework, neuroimaging studies provide some support to the idea that fearful eyes are salient, even if they are irrelevant to the task [13, 35, 36]. These studies focused on the response of the amygdala, a structure associated with emotional processing in the brain. For example, Whalen et al. [13] reported an increase in amygdala responses to fearful eyes relative to happy eyes, when presented in isolation and below the conscious threshold. The amygdala, also, showed elevated responses to fearful eyes relative to fearful mouth and neutral eyes when these were embedded in a canonical face [36]. Using evoked related responses with epileptic patients, it is reported that amygdala modulates early visual responses (i.e., ~100ms) to fearful versus neutral faces, even when the expression was irrelevant to the task [37]. Taken together, the data suggest that eyes–and especially fearful eyes (fearful faces)–drive emotion-specific neural responses, even if recognizing the expression is irrelevant to the task.

`The amygdala responses to fearful faces are primarily driven by LSF information in the image [38, 39]. LSF information is projected by the magnocellular pathway (from retina via thalamus to cortex). The magnocellular pathway is a relative fast visual route, conveying coarse description of the visual input, which is used to guide follow up processing [40–42]. Therefore, it is possible that the low-level attributes of expressive features, specifically their LSF power, underlie their perceptual saliency.

### Saliency of smiling mouth

The effect of the smiling mouth on perception and recognition has not received the same scrutiny as fearful eyes. Using the bubble method, as above, it has been shown that the mouth is important for recognising happy expressions [2]. Calder and colleagues [30], also, reported that it is easier to recognize happy expression from the bottom rather than the top part of the face. Accordingly, gaze patterns are biased towards, and fixated longer on the mouth region in happy expressions compared to all the other expressions [6, 31, 32, 34]. Nevertheless, participants dwelled longer on happy eyes than smiling mouth [32, 34]. The detection of an oddball expressive mouth, among neutral mouths, is superior to the detection of oddball expressive eyes [6]. This indicates that the mouth superiority effects are modulated by the expressive features. There was a larger advantage for a smiling mouth over a fearful mouth, but a reverse advantage for fearful eyes over happy eyes [6]. Like with fearful eyes, discriminating a joyful expression from a neutral expression involves longer dwell times on the mouth for both expressive faces [33].

A remaining question is what makes the smile capture attention? On the one hand, Calvo and colleagues [6], argued for a visual-level or perceptual explanation for the smiling mouth advantage [43, 44]. Calvo et al. [43] argued that smiling is categorically and perceptually distinct from non-smiling faces as it draws an individual’s attention to fixate initially on the mouth. This tendency is increased due to the presence of teeth [45], indicating that low-level visual features may primarily increase the perceptual saliency of the mouth. On the other hand, the affective account suggests that presence of smile can be used as heuristic to recognise and categorise the expression of happiness with efficiency and ease [46]. The mouth shape facilitates speed and response selection since there is a lack of competition from other facial expressions. The present study investigated whether the smile advantage would, also, be observed in a task that does not require the recognition of expression, which would help to explain the sources of its perceptual advantage.

### Expressive features and face configuration

If expressive features are perceptually salient due to their visual properties (e.g. power of LSF), and not due to their diagnostic value (e.g. fear is different compared to neutral as the former indicates danger), then the context in which the expressive features are presented would have little impact on their perception. Nummenmaa and Calvo [47] conducted a meta-analysis of search detection tasks, in which they compared the detection of an expressive feature in the context of canonical and non-canonical (e.g. inverted, scrambled features, features presented alone) faces. The results showed that the detection of a specific expression is only minimally affected by the facial context. In other words, violating the canonical configuration of faces led to a similar pattern as their canonical counterparts.

When violating the canonical configuration of faces (i.e. face inversion) the gaze is still biased toward the eyes [48]. However, there is a reduction in the spontaneous fixation on the eyes for inverted relative to the canonical configured faces [3, 48]. The recognition of expression from individual features (eyes or mouth region) or from inverted faces is compromised relative to the whole upright face [30]. But, the effects differ between expressions. The recognition of inverted fear is less accurate and slower than when fear is presented in a non-canonical configuration, while there is no inversion effect on the recognition of happiness [6, 49]. In summary, previous literature suggests that expressive features can capture attention on their own, although the effect is enhanced when they are presented in a context of a canonical face, especially for fearful eyes. This support at least some contribution, of the affective meaning of the features to their saliency.

### Current study

The aim of the current study was to revisit the hypothesis that expressive features are perceptually salient, which are based on bottom-up cues. As mentioned above, we aimed to test three predictions derived from the visual-based account: 1) expressive features would be salient even when the emotional information they convey is irrelevant to the task; and 2) the saliency of expressive features would not depend on their configuration (i.e., spatial relation to other features). In other words, the saliency of the expressive features would not be enhanced when they are presented in a canonical face configuration. Assuming only a canonical face configuration can present a ‘real’ emotion. 3) the saliency of a feature would relate to its low level visual properties. Specifically, the power of LSF in the features.

**Fig 1:**
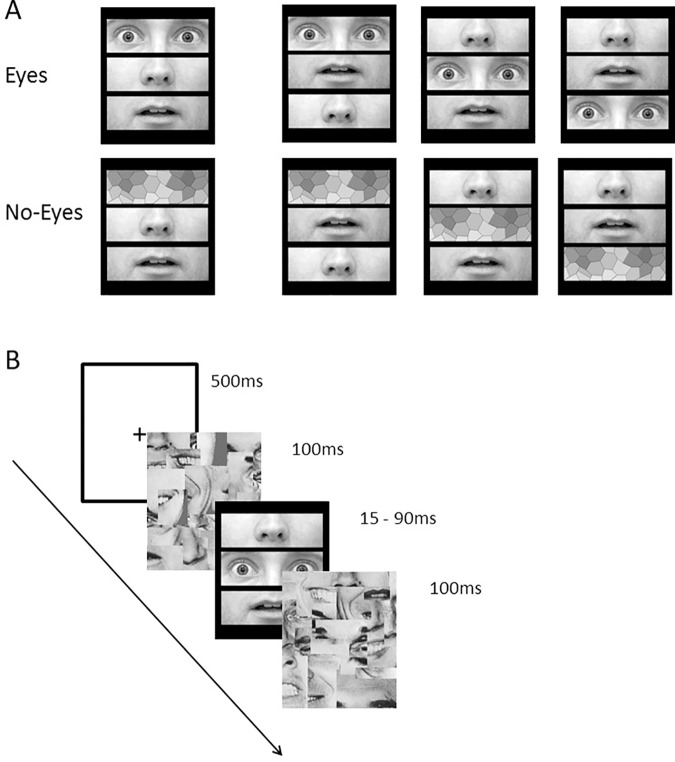
experiment 1 –Stimuli and trial procedure. A. The eight different stimuli types that were used in the experiments. B. The trial sequence. We note, that in the real experiment we used the faces of Ekman and Friesen series, here for descriptive purposes we provide an example of a face that is not part of this series. The individual in this manuscript has given written informed consent to publish these case details.

To test the first prediction, we used a feature detection task. Participants had to detect the presence of the eyes (experiments 1-3a) or mouth (experiment 3b). Importantly, the task was designed to ensure that the emotion of the feature/face was rendered as an irrelevant dimension. The task required a detection of a facial feature irrespective of its emotion. Thus, participants had to respond ‘yes’ when they saw eyes or mouth irrespective of their expressions. To test the second prediction, the target features were presented in a context of canonical or scrambled (non-canonical) configurations (Fig 1). To test the third prediction (section 5.2) we computed the LSF power in the target features and correlated it with the behavioural performances.

We applied Signal Detection Theory (SDT) to dissociate discriminability (*d*’) and response bias (*c*) associated with the detection of target features in different contexts (i.e. expression and facial configuration). *d’* is computed based on the difference between hit rates (correct detection of the eyes or mouth when they are present) and false alarms (false detection of the eyes or mouth when they were absent). Therefore, it provides an unbiased measure for bottom-up perceptual saliency. Criterion response bias, on the other hand, measures participants’ likelihood of making one response over the other. It reflects the top-down strategic component of the decision which is orthogonal to the ability to discriminate the presence of a target among noise [50]. We analysed the data using both subjects and items (faces) as random variables. This enabled us to generalize the findings beyond a specific set of participants or images.

Given that humans are excellent face detectors [51], the target stimuli were presented for brief duration [52, 53] with a backward and forward masks to avoid ceiling effects. The masks were created using a collage of facial parts, with no full feature presented [51] (Fig 1).

Experiments 1, 2 and 3A tested the ability to detect eyes and experiment 3B tested the ability to detect a mouth in canonical and non-canonical scrambled faces. Experiment 1 compared the detection of neutral and fearful eyes. Experiment 2 and 3A compared the detection of happy and fearful eyes. Experiment 3B compared the detection of mouth in happy or fearful expressions.

## Experiment 1: Detecting fearful and neutral eyes

Experiment 1 aimed to test whether fearful eyes are perceptually more salient than neutral eyes. It, also, aimed to test whether the saliency of fearful eyes depended on their relation to other facial features. In other words, would eyes presented in the context of canonical face be more salient? As mentioned above, if features are salient due to their visual properties, we expect that detection in the context of a canonical faces would not be superior than in the context of non-canonical configuration. Furthermore, we were specifically interested to assess whether the location of the eye target within the visual field had an impact on its detectability. We manipulated this latter variable in the context of the non-canonical, scrambled faces, where the target feature was moved in the visual field (e.g. top, middle and bottom; Fig 1A).

## Methods

### Participants

22 undergraduate students aged 19–25 years (*M* = 19.68 ± 1.33) from the University of Birmingham participated in this study for course credits. Data from two participants were lost due to technical errors. The experiment was run according to British Psychological Society ethical guidelines and was approved by the University of Birmingham ethical committee. All participants had normal or corrected-to-normal vision and signed an informed consent to take part in the study.

### Material

Stimuli were produced from 8 facial identities (nr, jj, mf, mo, pe, pf, sw, wf; Ekman & Friesen, [54]). Each identity exhibited a neutral and fearful expression. The stimuli were edited using an image manipulation program (GIMP2; www.gimp.org). Each face was zoomed in order to remove the hair and the external contours. The images were scaled to 200 x 195 pixels. Next, each face was divided into 3 horizontal rectangles with equal size (200 x 65 pixels): i) top: eyes + eyebrows, ii) middle: nose + cheeks and iii) bottom: the mouth + chin. These regions were cropped from the face and placed on a black background 3mm apart (Fig 1A). The stripes subtended a visual angle of 5.04 x 3.28°. The three horizontal lines were presented at a canonical configuration: eyes above nose above mouth or at a non-canonical configuration. There were three versions of the non-canonical configuration: i) eyes at the bottom: nose above mouth above eyes; ii) eyes in the middle: nose above eyes above mouth; and iii) eyes at top: eyes above mouth above nose (Fig 1A).

For the no-eye stimuli, we used the Hexagon ‘mosaic’ tiles filter (tile size: 30) to distort the information in the eyes rectangle. This ensured that the overall luminance and contrast was maintained across both conditions. However, in contrast to random noise, the filtering procedure meant that the no-eye stimuli did contained some of the structural information typically associated with eyes, which made the task relatively challenging. In order to further avoid ceiling effects, we degraded the stimuli presentation using backward and forward masks. The masks were created by extracting parts of facial features from the original stimuli and overlaid on each other to create a collage [51]. Importantly none of the extracted features comprised a full feature or a full eye (Fig 1B). Finally, we used root mean square to normalize the luminance (128) and contrast (85) across all stimuli including the masks.

### Procedure

The experiment had a nested within-subject design, with the following factors: expression (fear and neutral) and configuration (canonical and scrambled). The scrambled factor was further divided into three (nested) conditions based on the location of the eyes: top, middle, bottom. Each condition appeared with an equal probability, hence the ratio between canonical-to-all scrambled was 1:3. E-prime2.0 (https://www.pstnet.com/eprime.cfm) was used to realize the experiment and collect the responses.

An eye detection task was used. Participants were asked to press ‘z’ if they detected an eye, or ‘m’ if they did not (key-response mapping was counterbalanced across participants). Prior to the experiment, participants practiced the task over at least 72 trials (representing all possible stimuli type), in blocks of 20 trials. The practice had two aims: 1) to familiarize the participants with the task and stimuli set, 2) to establish individual target exposure duration. We adjusted the exposure duration individually, as there is individual variability in the efficiency and speed of sensory processing [55]. Therefore, perception was kept around the conscious threshold in order to avoid ceiling and floor effects [52, 53].

The practice started with relatively slow target exposure duration (i.e., 120ms) to enable supra-liminal perception. The duration was reduced gradually after each block, depending on the performance, until participants’ maintained around 70% detection accuracy. This was typically around 40-60ms, although there was large variability between participants.

A trial began with a fixation point for 500ms at the centre of the screen (Fig 1B). This was followed by a forward mask for 100ms. Subsequently, the target stimulus was presented with the specified target duration, followed by a backward mask for 100ms. Finally, a reminder screen, mapping the keyboards to the responses, was presented (e.g. ‘press the ‘Z’ for eyes and ‘M’ for no eyes’) in order to reduce memory demands. The order of the conditions was randomised. Overall, there were 256 stimuli (2(expression) x 4(configuration) x 2(eyes) x 8(faces) x 2(stimuli repetition)), equally distributed across all conditions, 16 per trial type. After every 32 trials, participants had a short break and received generic feedback about the overall accuracy of their performances in order to keep them motivated.

### Analysis

Trials with target presentation that deviated by 20ms or more from the intended presentation duration were removed from the analyses. The data were summarised and analysed twice, once treating the subjects as a random variable (subjA, N = 22), and a second using the faces as random variable (faceA, N = 8). We considered results as reliable only if an effect was found significant in both the subject- and item-based analyses.

Signal detection theory was used to dissociate the two components of the decision: sensory sensitivity to the presence of the eyes, denoted as the discriminability index (D-Prime or *d*’) and the cognitive decision process known as the criterion bias (*c*). For each participant/face and each condition, we computed the probability for a Hit response (correct detection of the eyes when they are present) and a false alarm (FA) response (false detection of the eyes when they were absent). Hit rate and false alarms were transformed into *Z* values. As zero and one rates do not have a normalized scores: Scores of 1 were replaced by 1-1/(number of trials per condition)*2, and zero by 1/(number of trials per condition)*2. The normalised scores of the hit and FA were subtracted within the same condition to obtain the d’ for that condition (*Z*(hit_1_)–*Z*(FA_1_). Higher *d’* indicates better discriminability index. *C* was computed as the ‘negative’ average of the normalized hit and FA responses (-0.5*(*Z*(hit_1_) + *Z*(FA_1_)). If *c* > 0, it would indicate very conservative approach to detect a target, whereas if *c* < 0, it would indicate very liberal approach to indicate that a target is present. In other words, the lower *c* means that participants were more likely to report that the target was present leading to higher false alarms but also higher hits [50].

We used a repeated measure ANOVA to compute the reliability of effects across subjects or items, using *d’* and *c* as dependent measures. We first tested the effects of expression (fear and neutral) and configuration (canonical and non-canonical). Subsequently, we focused on the different non-canonical configurations using a 2x3 design with the following factors: expression (fear and neutral) and target location in the scramble configuration (eyes top, eyes middle and eyes bottom). Greenhouse-Geisser corrections were used when reporting the reliability of the effects of the configuration manipulation.

## Results

The range of exposure duration was between 15–90 ms (*M* = 50.25 ± 16.74). We removed two trials due to errors in the presentation time. The overall accuracy of detecting the eyes was 66.8% ranging from 55–85% (9.7% std) at the subject level, and between 63 and 68% (1.7% std) at the item level. On average, the accuracy indicated that perception was around the conscious threshold. 14 out of the 352 (22 participants*16 trial types) data points, ~4% at the subject level analysis had boundaries values with a hit rate of one, or FA rate of zero; see Table 1 for the break down per condition. No data point had to be changed for the item analysis.

**Table 1:** Results of Experiment 1, detecting fearful versus neutral eyes (N = 22).

	Random variable	Hits	FA	D’	C
		M (Std), BC	M (Std), BC	M (Std)	M (Std)
**Fearful**					
**Canonical**	Subject	0.80 (0.21), 4)	0.44 (0.27), 1	1.28 (1.52)	-0.47 (0.46)
	*Item*	0.80 (0.03)	0.44 (0.05)	1.01 (0.14)	-0.39 (0.05)
**Scrambled overall**	Subject	0.86 (0.09)	0.38 (0.23)	1.58 (0.95)	-0.43 (0.46)
	*Item*	0.86 (0.02)	0.38 (0.02)	1.40 (0.17)	-0.35 (0.11)
**Scr-bottom**	Subject	0.83 (0.15), 2	0.32 (0.27), 1	1.68 (1.12)	-0.27 (0.63)
	*Item*	0.83 (0.03)	0.32 (0.03)	1.42 (0.13)	-0.25 (0.09)
**Scr-middle**	Subject	0.92 (0.08),8	0.29 (0.25),1	2.30 (1.12)	-0.45 (0.57)
	*Item*	0.93 (0.02)	0.29 (0.04)	2.04 (0.24)	-0.45 (0.12)
**Scr-top**	Subject	0.83 (0.15),1	0.53 (0.25)	1.06 (1.02)	-0.59 (0.52)
	*Item*	0.83 (0.05)	0.53 (0.04)	0.88 (0.29)	-0.52 (0.07)
**Neutral**					
**Canonical**	Subject	0.72 (0.20)	0.57 (0.26)	0.47 (0.99)	-0.43 (0.55)
	*Item*	0.71 (0.17)	0.57 (0.12)	0.62 (0.15)	-0.38 (0.22)
**Scrambled overall**	Subject	0.66 (0.18)	0.42 (0.17)	0.71 (0.56)	-0.08 (0.48)
	*Item*	0.65 (0.15)	0.42 (0.03)	0.43 (0.24)	-0.10 (0.09)
**Scr-bottom**	Subject	0.53 (0.23)	0.34 (0.14)	0.57 (0.84)	0.20 (0.52)
	*Item*	0.53 (0.08)	0.34 (0.05)	0.50 (0.14)	0.18 (0.17)
**Scr-middle**	Subject	0.65 (0.22),2	0.33 (0.18)	1.00 (0.75)	0.07 (0.58)
	*Item*	0.65 (0.08)	0.33 (0.04)	0.85 (0.19)	0.02 (0.13)
**Scr-top**	Subject	0.79 (0.19)	0.58 (0.27)	0.67 (0.87)	-0.59 (0.66)
	*Item*	0.79 (0.05)	0.58 (0.09)	0.61 (0.21)	-0.50 (0.18)

Index: M, mean, std, standard deviation, BC, number of participants having performing at ceiling (Acc = 100% of detecting eyes) or floor (Acc = 0%, indicating noise as eyes), no values indicate that no participant showed boundary performance.

**Fig 2:**
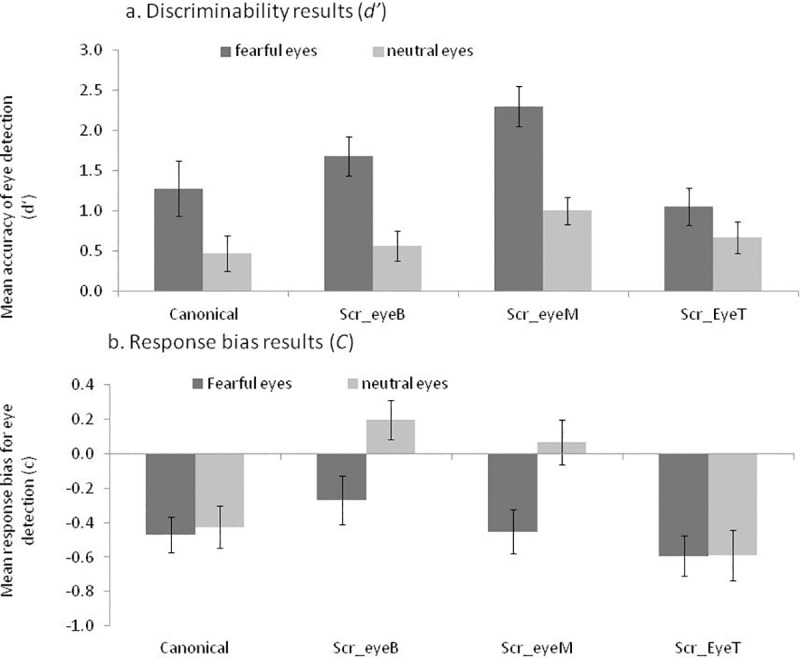
Experiment 1 –Results. The charts represent the d’ prime (A) and response bias (B) results, see details Table 1. Scr, scrambled configuration, B eyes on the bottom stripe; M, eyes on the middle stripe, T, eyes on the top stripe.

Across conditions, participants could clearly discriminate between eyes and no-eyes, as *d’* was reliably higher than zero (subjA: *F* (1, 19) = 36.62, *p* < .0001, *η*_*p*_^2^ = .65; faceA: *F* (1, 7): 46, *p* < .0001, *η*_*p*_^2^ = .98). However, participants had overall a clear bias to indicate that eyes were present, *c* was reliably below zero (subjA: *F* (1, 19) = 14.31, *p* < .0001, *η*_*p*_^2^ = .43; faceA: *F* (1, 7) = 100.24, *p* < .0001, *η*_*p*_^2^ = .93). The results broken down by conditions are provided in Table 1. For completeness and clarity, we also provided the accuracy results in the table. To aid clarification, Fig 2 displayed charts of *d’* and *c* based on the subject-based analysis.

### Discriminability index (d’) analysis

We, first, examined the effects of expression (fear and neutral) and configuration (canonical and non-canonical) on ability to detect the eyes. In this analysis, we collapsed across the three versions of the non-canonical scrambled faces. We computed two ANOVAs, using subjects (*n* = 20), or items (*n* = 8) as random variables. In both analyses, we observed that detection of fearful eyes was better than the detection of neutral eyes (main effect of expression, subjA: *F* (1, 19) = 10.23, *p* = .005, *η*_*p*_^2^ = .35; faceA: *F* (1, 7) = 400.33, *p* < .0001, *η*_*p*_^2^ = .98). A main effect of configuration showed that the scrambled condition was associated with better eye detection than the canonical condition (subjA: *F* (1, 19) = 4.58, *p* < .046, *η*_*p*_^2^ = .19; faceA: *F* (1, 7) = 15.36, *p* = .006, *η*_*p*_^2^ = .69). There was no significant interaction between expression and facial configuration (*p* > .13).

We next focused only on the scrambled condition. As mentioned above, in the scrambled condition the eyes were located at the top, middle or bottom of the image. Therefore, analysis computed the effects of expression (fear and neutral) and eye location (eyes top, middle and bottom) on the ability to detect the eyes. There was a main effect of expression, with fearful eyes detected better than neutral eyes (subjA: *F* (1, 19) = 16.1, *p* = .001, *η*_*p*_^2^ = .46; faceA = *F* (1, 7) = 182.1, *p* < .0001, *η*_*p*_^2^ = .96). There was a main effect of the location of the eyes within the image (subjA: *F* (2, 38) = 15.7, *p* < .0001, *η*_*p*_^2^ = .45; faceA: *F* (2, 14) = 88.7, *p* < .001, *η*_*p*_^2^ = .92). There was also an interaction between expression and configuration (subjA: *F* (1.38, 26.17) = 5.71, *p* = .016, *η*_*p*_^2^ = .23; faceA: *F* (2, 14) = 27.84, *p* < .0001, *η*_*p*_^2^ = .79).

To better understand the source of this interaction we analysed responses to each expression separately and followed it up with t-test if needed. We used Bonferroni correction to report the reliability of *t*-tests (corrected *p* value: 0.05/12 = 0.0042).

For fearful expression, there was a main effect of the eyes location (subjA: *F* (2, 38) = 22.1, *p* < .0001, *η*_*p*_^2^ = .54; faceA: *F* (2, 14) = 71.3, *p* < .0001, *η*_*p*_^2^ = .91). Not surprisingly, a follow-up t-tests showed that fearful eyes were detected with more precision when they were presented at the middle compared with the bottom (subjA: *t* (19) = 6.21, *p* < .0001; faceA: *t* (7) = 6.9, *p* < .0001) and the top (subjA: *t* (19) = 6.2, *p* < .0001; faceA: *t* (7) = 9.5, *p* < .0001). Eyes presented at the bottom were also better detected than those presented at the top (subjA: *t* (19) = 2.67, *p* = .015 (uncorrected); faceA: *t* (7) = 7.3, *p* < .0001).

For the neutral expression, there was no reliable effect of the eye location in the subject-based analysis (subjA: *F* (2, 38) = 2.23, *p* = .122, *η*_*p*_^2^ = .10), though there was a reliable effect in the face/item based analysis (faceA: *F* (1.52, 10.66) = 14.44, *p* = .002, *η*_*p*_^2^ = .67). Follow-up t-tests showed that like with the fearful expression, eyes in the middle were detected better than eyes at the bottom (faceA: *t* (7) = 4.36, *p* = .003) and at the top (faceA: *t* (7) = 5.1, *p* = .001). There was no reliable detection difference for neutral eyes presented at the top or the bottom (faceA: *t* (7) = 1.64, *p* = .14).

These follow-up analyses showed that the expression by configuration interaction arose from the fact that the location of the eyes had a stronger impact on the detection of fearful than neutral eyes. Though for both expressions, eyes at the middle were detected better than eyes at the bottom or the top.

In summary, participants showed higher sensitivity to fearful eyes than neutral eyes and surprisingly, there was a higher sensitivity to eyes in the scrambled rather than canonical configuration. The latter effect was primarily driven by the increased detection for the eyes being presented in the middle row, an effect that was stronger for fearful eyes.

### Criterion bias (c) analysis

Results are presented in Table 1 and Fig 2B. We first examined the effects of expression (fear and neutral) and configuration (canonical and non-canonical) on ability to detect the eyes. In this analysis, we collapsed across the three versions of the non-canonical scrambled faces. We observed an overall higher bias to report eyes present for fearful than neutral expressions (main effect of expression, subjA: *F* (1, 19) = 11.59, *p* = .003, *η*_*p*_^2^ = .38; faceA: *F* (1, 7) = 6.32, *p* = .04, *η*_*p*_^2^ = .47). A main effect of configuration showed that the tendency to report eye present was higher in the canonical than the scrambled condition (subjA: *F* (1, 19) = 5.41, *p* < .031, *η*_*p*_^2^ = .22; faceA: *F* (1, 7) = 10.31, *p* = .015, *η*_*p*_^2^ = .60). There was also a significant interaction between expression and facial configuration (subjA: *F* (1, 19) = 7.65, *p* = .012, *η*_*p*_^2^ = .29; faceA: *F* (1, 7) = 42.36, *p* < .0001, *η*_*p*_^2^ = .86).

To better understand this interaction, we next compared the effect of face configuration on each expression separately. Face configuration did not change response bias for fearful expressions (all *p*s > .24), but there was a stronger bias to report eyes in canonical than scrambled neutral face faces (subjA: *t* (19) = 3.06, *p* = .006; faceA: *t* (7) = 5.36, *p* = .001).

We next tested the effect of the target (eye/no eyes) location on response bias using a 2 (expression) by 3 (eye location in the scrambled face) repeated measured ANOVA. Fearful expressions were associated with higher tendency to report eyes present compared with neutral (subjA: *F* (1, 19) = 29.04, *p* < .0001, *η*_*p*_^2^ = .60; faceA: *F* (1, 7) = 70.3, *p* < .0001, *η*_*p*_^2^ = .91). There was also a main effect of target location (subjA: *F* (2, 38) = 19.39, *p* < .0001, *η*_*p*_^2^ = .51; faceA: *F* (2, 14) = 58.6, *p* < .0001, *η*_*p*_^2^ = .89). These two latter main effects should be interpreted in the light of a reliable crossover interaction (subjA: *F* (2, 38) = 8.61, *p* = .001, *η*_*p*_^2^ = .31; faceA: *F* (2, 14) = 11.9, *p* = .001, *η*_*p*_^2^ = .63).

To follow up on the interactive effect of target location and expression on response bias, we computed separate ANOVAs for each expression, followed up by t-tests which were corrected for multiple comparisons, as stated above for *d’*.

For fearful expressions, response bias was affected by the location of the targets (subjA: *F* (2, 38) = 3.29, *p* = .048, *η*_*p*_^2^ = .14; faceA: *F* (2, 14) = 16.43, *p* < .0001, *η*_*p*_^2^ = .70). The bias increased as the location of eyes/no eyes was higher in the image, though the t-tests suggest that these effects had weak reliability, as most did not survive the correction for multiple comparisons *p* < .004 (fear eye Bottom–eye Top—subjA: *t* (19) = 2.21, *p* = .04; faceA: *t* (7) = 11.39, *p* < .0001; fear eye Bottom vs. eye Middle—subjA: *t* (19) = 2.03, *p* = .056; faceA: *t* (7) = 3.62, *p* = .008).

For the neutral expression, response bias was also affected by the location of the targets (subjA: *F* (2, 38) = 34.58, *p* < .0001, *η*_*p*_^2^ = .64; faceA: *F* (2, 14) = 37.11, *p* < .0001, *η*_*p*_^2^ = .84). Follow-up analysis showed that the response bias to neutral eyes showed a similar pattern to the one observed to fearful expressions. Participants showed higher tendency to report eyes when the targets were presented at the top of the face (fear eye Top vs. Middle—subjA: *t* (19) = 5.27, *p* < .0001; faceA: *t* (7) = 6.05, *p* = .001; fear eye Middle–Bottom—subjA: *t* (19) = 1.87, *p* = .077; faceA: *t* (7) = 3.53, *p* = .01).

These follow-up analyses showed that the expression by configuration interaction arose from the fact that the location of the eyes had a stronger impact on response bias for neutral than fearful expressions. Though for both expressions the higher the location of the target stimuli was the more biased were participate to indicate a present of eyes (top > middle > bottom).

## Discussion

The results of Experiment 1 demonstrated that under the same degraded viewing conditions, fearful eyes are detected with more precision than neutral eyes. Surprisingly, better detection was observed when the canonical configuration was violated. The results demonstrated that presenting eyes in a context of a canonical face, i.e. implying ‘real’ emotions, did not increase the perceptual saliency of the feature, but decreased it. This finding support the visual-based hypothesis, where saliency is not derived from the emotional value but the visual properties of the stimulus. This observation challenges the affective-based account, as we assumed that violating the face configuration reduces its emotional value.

Why was there an advantage for detecting eyes in non-canonical configuration? One explanation could be that holistic face processing may inhibit identification of facial features due to the activation of the ‘full face’ representation. However, this explanation is questionable, as previous evidence suggests that processing of individual feature is facilitated, not degraded, when presented in a context of a canonical compared to a jumbled face [56]. Similarly, at a neuronal level, it is suggested that holistic perception (grouping of features by gestalt laws) is enhanced rather than suppressed the activation of the individual features [57], with an automatic spread of activation to all features that can be grouped by gestalt criteria [58]. Thus, it is unlikely that the canonical context have led to the suppression of responses to the individual features.

Alternatively, we note that there may be a more trivial explanation for this result pattern. When breaking down the scrambled condition, the data suggested that the overall advantage of non-canonical configuration was primarily driven by the trials in which the eyes were presented at fixation. Thus, the advantage of non-canonical configuration is likely to be confounded by the spatial location of the eyes relative to fixation. This effect was facilitated for fearful eyes, suggesting that their visual properties (proximity to fixation) and not their emotional values (being part of a real face) drove the detection precision results.

Interestingly, the criterion bias was affected by the facial configuration in the expected way, with a stronger tendency to report the presence of the eyes when the features were presented in a canonical configuration, or when the eyes were presented at the top, as common in canonical configuration (see General Discussion, for more elaborate theoretical consideration). This also suggests that affective processing and prior knowledge contributed to the perception of the salient features. this contribution is implemented as a top-down mechanism; but this does not directly impact the precision of sensory processing.

## Experiment 2: Detection fearful vs. happy eyes

Experiment 2 asked whether the advantage of fearful eyes is driven by their emotional expression. Hence, would any emotional feature be more salient than a neutral feature or do fearful eyes have something special? To assess this, we compared the detection of fearful eyes with happy eyes. We used only one scrambled condition (nose above mouth above eyes (Fig 1A). This scrambled version was selected for two reasons: 1) to avoid confounding the configuration manipulation with the proximity of the target feature to the fixation point and 2) to ensure maximum deviation from a canonical face.

## Methods

### Participants

15 undergraduate female students aged 18–20 years (*M* = 20) from the University of Birmingham participated in this study for course credits. The experiment was run according to the British Psychological Society ethical guidelines, which was approved by the University of Birmingham. All participants had normal or corrected sight and provided informed consent to take part in the study.

### Material, procedure and analysis

The materials were similar to the one used in experiment 1 except that happy expressions replaced the neutral expressions and mosaic with quadratic filter was used instead of hexogen to maskout the eyes. In addition, we also added 2 other identities from the Ekman and Friesen Series [54], to have the 10 total original identities. The procedures were identical to experiment 1. The practice trial had 5 blocks of faces, with 20 stimuli per condition. For the real experiment, there were 320 trials, each face stimulus was presented in four blocks with 80 trials each. There were resulting in 80 trials per condition (2 x 2), half presented with eyes and half without eyes (filtered eyes). The same analysis as experiment 1 was used.

## Results

The range of exposure duration was 35 to 130ms (*M* = 88 ± 21.7). We removed 121 trials due to error in the target presentation time. The overall accuracy of detecting the eyes was 72%, ranging from 55–87% (8% std) at the subject level, and between 63 and 79% (5.5% std) at the item level. As intended, the accuracy indicated that perception was around the conscious threshold. Eight out of the 120 (15 participants*8 trial types) data points (6.6%) at the subject level analysis, had boundaries values, see Table 2 for the break down per condition. No data point had to be changed for the item analysis.

**Table 2:** Results of Experiment 2, detecting fearful versus happy eyes.

	Random variable	Hits	FA	D’	C
		M (Std), BC	M (Std), BC	M (Std)	M (Std)
**Fearful**					
**Canonical**	Subject	0.81 (0.20),3	0.31 (0.14)	1.68 (0.88)	-0.30 (0.44)
	*Item*	0.91 (0.04)	0.31 (0.18)	1.94 (0.55)	-0.42(0.28)
**Scrambled**	Subject	0.74 (0.20),3	0.28 (0.15)	1.46 (0.81)	-0.08 (0.47)
	*Item*	0.84(0.07)	0.28 (0.11)	1.63 (0.43)	-0.21 (0.23)
**Happy**					
**Canonical**	Subject	0.70 (0.21),2	0.47 (0.15)	0.78 (0.81)	-0.29 (0.49)
	*Item*	0.77 (0.12)	0.47 (0.22)	0.85(0.73)	0.02(0.38)
**Scrambled**	Subject	0.64 (0.17)	0.30 (0.17)	0.97 (0.64)	0.11 (0.39)
	*Item*	0.66 (0.19)	0.37 (0.28)	0.94 (0.74)	-0.35 (0.37)

Index: M, mean, std, standard deviation, BC, number of participants having performing at ceiling (Acc = 100% of detecting eyes) or floor (Acc = 0%, indicating noise as eyes), no values indicate that no participant showed boundary performance.

Across conditions, participants could clearly discriminate between eyes and no-eyes, as *d’* was reliably higher than zero (subjA: *F* (1,14) = 78.8, *p* < .0001, *η*_*p*_^2^ = .85; faceA: *F* (1,9) = 198, *p* < .0001, *η*_*p*_^2^ = .96). In contrast to experiment 1, participants overall had less clear bias to indicate that eyes were present, *C* was only reliably below zero for the item analysis (subjA: *F* (1,14) = 2.74, *p* = .12, *η*_*p*_^2^ = .16; faceA: *F* (1,9) = 16.8, *p* = .003, *η*_*p*_^2^ = .65). The results broken down by conditions are provided in Table 2.

### Discriminability index (d’) analysis

We examined the effects of expression (fear, happy) and configuration (canonical, non-canonical) on ability to detect the eyes. We computed two ANOVAs, using subjects (*n* = 15), or items/faces (*n* = 10) as random variables, with *d’* and *c* as the dependent variable. In both analyses, we observed that detection of fearful eyes was better than the detection of happy eyes (main effect of expression, subjA: *F* (1, 14) = 9.5, *p* = .008, *η*_*p*_^2^ = .40; faceA: *F* (1, 9) = 30.1, *p* < .0001, *η*_*p*_^2^ = .77). There was no effect of configuration (all *p*s > .60), nor did configuration interact with expression (subjA: *F* (1, 14) = 3.8, *p* = .07, *η*_*p*_^2^ = .22; faceA: *F* (1, 9) = 1.2, *p* < .30, *η*_*p*_^2^ = .11).

### Criterion bias (c) analysis

Repeated measured ANOVA with 2(expression) by 2(configuration) factors was used to test the effects on the criterion bias. The tendency to report the presence of the eyes was higher in the fearful expression, though this was only reliable in the item based analysis (subjA: *F* (1, 14) = 1.5, *p* = .24, *η*_*p*_^2^ = .10; faceA: *F* (1, 9) = 8.3, *p* = .018, *η*_*p*_^2^ = .48). Participants were more likely to report the presence of the eyes in canonical faces than non-canonical faces, though this effect was reliable in the subject-based analysis and there was only a trend observed in the item-based (subjA: *F* (1, 14) = 12.1, *p* = .004, *η*_*p*_^2^ = .46; faceA: *F* (1, 9) = 4.5, *p* = .062, *η*_*p*_^2^ = .34). The interaction was not reliable (all *p*s > .3).

## Discussion

The results showed that participants had higher precision in detecting fearful eyes than happy eyes, irrespective of the facial configuration. The superior detection of fearful eyes replicated the results of experiment 1, showing a robust advantage for detecting fearful than non-fearful eyes. Although in experiment 1, we observed a superior ability to detect eyes in scrambled faces, experiment 2 does not reliably show the effect of configuration on the ability to detect eyes. This difference is likely to reflect the fact that the advantage of feature detection in the scrambled faces, observed in experiment 1, was confounded with the location of the target feature in the visual field (targets at fixation were detected better). Experiment 2 presented the faces in the canonical and scrambled version at equal distance from fixation which eliminated the advantage of the non-canonical configuration. Taken together, the results of both experiments suggest that eyes were detected better with fearful rather than non-fearful expressions. This supports the visual-based account for the saliency of expressive features.

Like in experiment 1, the analysis of the criterion biased showed a tendency for an overall bias to report the presence of the eyes in the context of canonical faces. This suggests that the facial context influences response bias, but not the discrimination. Fearful expression tended to increase response bias to indicate the eyes being present, but this effect was only reliable at the item-based analysis. This implies that the fearful expressions of the other features, or the activation of the entire face had some impact on top-down decision that eyes were present.

One potential limitation of experiment 1 and 2 is that the type of filter (i.e., mosaic filter) used to create the no-eyes condition affected the high spatial frequency information, while maintaining the low spatial frequency properties (e.g. two dark spots within a brighter surrounding; see Fig 1A). It has been shown that responses to fearful expression in emotional brain regions (amygdala) are driven by LSF [59]. Thus, it may be the filter that drove the results of the criterion bias. Therefore, we applied a different filter which distorted information across all frequency bands in experiment 3 (Fig 2). We further wanted to test whether the visual-based account for the saliency of expressive features could also account for the priority of the smiling mouth.

## Experiment 3: Detecting fearful eyes and mouth versus happy eyes and mouth

Experiment 1 and 2 showed that fearful eyes are more salient than happy and neutral eyes. However, eyes are not the only facial features that provide signals supporting the decoding of expressions. As mentioned in the introduction, it has been shown that the mouth is essential to detect happiness [2]. Therefore, experiment 3 tested whether the saliency of the eyes or mouth depends on its expression. In this experiment, participants were asked to detect eyes (Exp 3A) or mouth (Exp 3B) in separate blocks. Experiment 3A, replicated experiment 2, but used a different filter for the eyes. In addition, we fixed the exposure duration rather than estimating it for each participant in order to avoid confounding the exposure duration with the task requirement. Based on the results of experiments 1 and 2, and the previous literature, we predicted that: 1) fearful eyes would be detect better than happy eyes; 2) happy mouth would be detected better than fearful mouth; 3) eyes in general would be detected better than mouth; and finally, 4) there would be no effect of facial configuration on feature detectability. Nevertheless, we expected configuration to affect the criterion bias, by increasing eye/mouth present responses for canonical faces.

## Methods

### Participants

20 undergraduate students (18 females, *M* = 19.5) from the University of Birmingham participated in this study for course credits. The experiment was run according to the British Psychological Society ethical guidelines, which was approved by University of Birmingham ethics committee. All participants had normal or corrected sight and provided informed consent to take part in the study.

### Material, procedure and analysis

**Fig 3:**
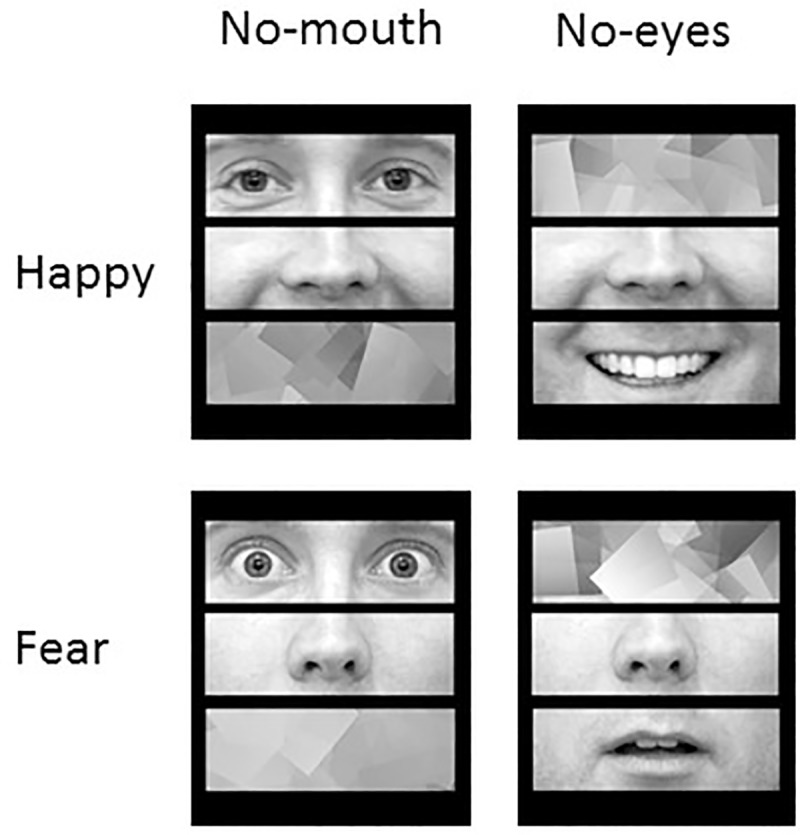
Experiment 3 –Stimuli example. An example of happy feature expressions and their filtered version. For descriptive purposes we provide an example of a face that is not part of this series. The individual in this manuscript has given written informed consent to publish these case details.

We used the same stimuli set as experiment 2. For the non-eyes/non-mouth stimuli, we used a cubism filter (tile size of 25 and saturation of 3; Fig 3). The eye present stimuli used in experiment 3A were identical to the ones used in experiment 2, but we used a different filter for the non-eye stimuli. Experiment 3B required the participants to detect the mouth. We also change the non-canonical configuration such that in the scrambled version mouth was at the top, above the eyes above nose. This configuration ensured that the distance of the target (mouth) from fixation is the same in the canonical and the scrambled versions. The trial procedure was identical to experiment 1 and 2, except that the exposure duration was fixed across all participants to 60ms.

Participants completed two separate tasks: detect eyes or detect mouth (Fig 2B and 2C). The number of trials was equally distributed across the conditions. There was a total of 320 trials in each experiment. In experiment 3A, half of the participants completed an additional number of 80 trials (half with eyes and half with no eyes) due to a programming error. We included these extra trials in the analysis. The tasks were run in separate blocks and the order was counterbalanced across participants. We used the ‘z’ and ‘m’ keys to report the presence and absence of a feature, counterbalanced across participants. To reduce cross-over interference, the response key for target was switched between the eyes and mouth tasks. We conducted the same analyses protocols as experiment 1 and 2, focusing on the two components of decision as extracted by SDT.

## Results

Overall, we removed 16 trials from the analysis in experiment 3A (eye detection) due to error in presentation time which was 20msec longer than 60msec. The overall accuracy of detecting the eyes was 76%, ranging from 58–87% (7% std) at the subject level, and between 67 and 80% (4% std) at the item level. As intended, the accuracy indicated that perception was around the conscious threshold. Eleven out of the 160 (20 participants*8 trial types) data points, 6.9% at the subject based analysis had boundaries values, see Table 2 for the break down per condition. No data point had to be changed for the item analysis.

**Fig 4:**
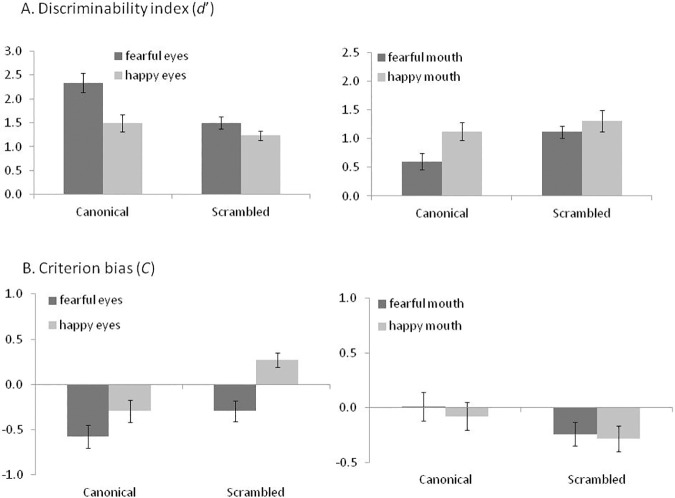
Experiment 3 –Results. The charts represent the d’ prime (A) and response bias (B) results, see details Table 3.

Like the previous studies, across conditions, participants could clearly discriminate between eyes and no-eyes, as d’ was reliably higher than zero (subjA: *F* (1, 19) = 209.4, *p* < .0001, *η*_*p*_^2^ = .92; faceA: *F* (1, 9) = 361.9, *p* < .0001, *η*_*p*_^2^ = .98). Like in experiment 2, participants had overall less clear bias to indicate that eyes were present, *c* was only reliably below zero for the item analysis (subjA: *F* (1, 19) = 3.8, *p* = .07, *η*_*p*_^2^ = .14; faceA: *F* (1, 9) = 14.4, *p* = .004, *η*_*p*_^2^ = .66). The results broken down by conditions are provided in Table 3A and Fig 4A.

**Table 3:** Results of Experiment 3, detecting fearful and happy eyes; fearful and happy mouth.

	Random variable	Hits	FA	D’	C
		M (Std), BC	M (Std), BC	M (Std)	M (Std)
**Fearful-eye**					
**Canonical**	Subject	0.94 (0.05),6	0.31 (0.19)	2.34 (0.80)	-0.57 (0.36)
	*Item*	0.94 (0.03)	0.30 (0.10)	2.18 (0.44)	-0.55 (0.16)
**Scrambled**	Subject	0.86 (0.12),2	0.26 (0.18),1	2.07 (0.89)	-0.20 (0.51)
	*Item*	0.85 (0.08)	0.25 (0.10)	1.81 (0.34)	-0.19 (0.28)
**Happy-eye**					
**Canonical**	Subject	0.82 (0.14),1	0.35 (0.21)	1.50 (0.44)	-0.29(0.54)
	*Item*	0.81 (0.10)	0.34 (0.11)	1.06 (0.55)	-0.25(0.29)
**Scrambled**	Subject	0.62 (0.17)	0.24 (0.18),1	1.23 (0.58)	-0.27 (0.56)
	*Item*	0.61 (0.18)	0.23 (0.05)	1.39 (0.48)	0.21 (0.28)
**Fearful-mouth**					
**Canonical**	Subject	0.60 (0.22)	0.40 (0.23)	0.60 (0.63)	0.01 (0.58)
	*Item*	0.60 (0.16)	0.40 (0.13)	0.55 (0.63)	.00 (0.24)
**Scrambled**	Subject	0.76 (0.14)	0.39 (0.20)	1.12 (0.47)	-0.24 (0.48)
	*Item*	0.76 (0.11)	0.39 (0.14)	1.08 (0.58)	-0.23 (0.26)
**Happy-mouth**					
**Canonical**	Subject	0.71 (0.22)	0.33 (0.20)	1.12 (0.70)	-0.08 (0.56)
	*Item*	0.71 (0.14)	0.33 (0.12)	1.05 (0.66)	-0.07 (0.18)
**Scrambled**	Subject	0.80 (0.14)	0.39 (0.24),1	1.31 (0.83)	-0.28 (0.52)
	*Item*	0.80 (0.16)	0.39 (0.11)	1.22 (0.61)	-0.32 (0.29)

Index: M, mean, std, standard deviation, BC, number of participants having performing at ceiling (Acc = 100% of detecting eyes and correctly rejecting noise). No participant showed a floor effect. No values indicate that no participant showed boundary performance.

Overall, we removed 7 trials from the analysis in experiment 3B (mouth detection) due to error in presentation time which was 20msec longer than 60msec. The overall accuracy of detecting the mouth was 67%, ranging from 52–78% (8% std) at the subject level, and between 57 and 72% (5.8% std) at the item/face level. As intended, the accuracy indicated that perception was around the conscious threshold. One out of the 160 (20 participants*8 trial types) data points, 0.6% at the subject based analysis had boundaries values, see Table 2 for the break down per condition. No data point had to be changed for the item analysis. Like the other experiments, we avoided floor and ceiling effects, participants could clearly discriminate between mouth and no-mouth, as *d’* was reliably higher than zero (subjA: *F* (1, 19) = 83.7, *p* < .0001, *η*_*p*_^2^ = .81; faceA: *F* (1,9) = 76.4, *p* < .0001, *η*_*p*_^2^ = .89). Like in experiment 2 and 3A, participants overall had less clear bias to indicate that mouth was present, *C* was only reliably below zero for the item analysis (subjA: *F* (1, 19) = 1.88, *p* = .19, *η*_*p*_^2^ = .09; faceA: *F* (1,9) = 15.2, *p* = .004, *η*_*p*_^2^ = .63). The results broken down by conditions are provided in Table 3B and Fig 4B.

### Discriminability index (d’) analysis

We first examined the effects of task (detect eye, mouth), expression (fear, happy) and configuration (canonical, non-canonical) on ability to detect an expressive feature. We computed two ANOVAs, using subjects (*n* = 20), or items/faces (*n* = 10) as random variables, using *d’* as the dependent variable. We observed a clear effect of task in both analyses (subjA: *F* (1, 19) = 37.9, *p* < .0001, *η*_*p*_^2^ = .66; faceA: *F* (1, 9) = 16.5, *p* = .003, *η*_*p*_^2^ = .65), showing that participants were overall better at detecting eyes than mouth. Fearful features were overall better detected than happy features, though this effect was only reliable at the subject level (subjA: *F* (1, 19) = 14.62, *p* = .001, *η*_*p*_^2^ = .44; faceA: *F* (1, 9) = 1.8, *p* = .22, *η*_*p*_^2^ = .17). The configuration of the features did not affect the results across conditions (all *p*s > .50). However, the task interacted with expression (subjA: *F* (1, 19) = 36.1, *p* < .0001, *η*_*p*_^2^ = .66; faceA: *F* (1, 9) = 34, *p* < .0001, *η*_*p*_^2^ = .79); and with the configuration manipulation (subjA: *F* (1, 19) = 10.2, *p* = .005, *η*_*p*_^2^ = .35; faceA: *F* (1, 9) = 6.9, *p* = .027, *η*_*p*_^2^ = .44). The three ways interaction was not reliable. As the task interacted with expression or configuration, we unpacked the 2-way interactions by computing a separate analysis for each task.

#### Experiment 3A: Eye detection

Replicating the results of experiment 1 and 2 fearful eyes were better detected than happy eyes (subjA: *F* (1, 19) = 41.7, *p* < .0001, *η*_*p*_^2^ = .69; faceA: *F* (1, 9) = 24.7, *p* = .001, *η*_*p*_^2^ = .73). The feature configuration also affected the ability to detect eyes (subjA: *F* (1, 19) = 5.3, *p* = .032, *η*_*p*_^2^ = .22; faceA: *F* (1, 9) = 12.2, *p* = .007, *η*_*p*_^2^ = .58). Unlike Experiment 1 and the pattern of results of experiment 2, participants were better at detecting eyes in the context of canonical than non-canonical faces in the current experiment. Expression and configuration did not interact (all *p*s > .90).

#### Experiment 3B: Mouth detection

Happy mouth was better detected than fearful mouth, but the effect was only reliable in the subject level analysis (subjA: *F* (1, 19) = 11.8, *p* = .003, *η*_*p*_^2^ = .38; faceA: *F* (1, 9) = 2.05, *p* = .19, *η*_*p*_^2^ = .19). Face configuration had a marginal effect (subjA: *F* (1, 19) = 8.22, *p* = .01, *η*_*p*_^2^ = .30; faceA: *F* (1, 9) = 3.65, *p* = .088, *η*_*p*_^2^ = .29). This showed that mouth detection in the scrambled condition was superior to the canonical configuration condition. Expression and configuration did not interact (all *p*s > .16).

In summary, eye detection was easier than mouth detection. Fearful eyes were detected better than happy eyes, but happy mouth was detected better than fearful mouth. Eye detection in the canonical configuration was superior to the non-canonical configuration, while mouth detection was superior in the scrambled configuration (see section 2.3, the discussion of experiment 1 on potential interpretations for this interaction pattern).

### Criterion bias (c) analysis

We started by examining the effects of task (eye and mouth), expression (fear and happy) and configuration (canonical and non-canonical) on response bias to indicate the presence of an expressive feature. We computed two ANOVAs, using subjects (*n* = 20), or items/faces (*n* = 10) as random variables, using *C* as the dependent variable. In both analyses, there was no reliable effect of task (all *p*s > .48). There was a stronger bias to report the fearful than the happy features, though this effect was only reliable at the subject level (subjA: *F* (1, 19) = 33.27, *p* < .0001, *η*_*p*_^2^ = .64; faceA: *F* (1, 9) = 4.5, *p* = .06, *η*_*p*_^2^ = .33). The configuration of the features affected the results across conditions, though this effect was also only reliable at the subject level (subjA: *F* (1, 19) = 6.27, *p* = .022, *η*_*p*_^2^ = .25; faceA: *F* (1, 9) = 4.3, *p* = .068, *η*_*p*_^2^ = .32). This showed that overall, participants were more likely to report the presence of an expressive feature in the canonical configuration than in the scrambled condition. However, the task interacted with expression (subjA: *F* (1, 19) = 29.25, *p* < .0001, *η*_*p*_^2^ = .61; faceA: *F* (1, 9) = 17.4, *p* = .002, *η*_*p*_^2^ = .66); and task interacted with the configuration manipulation (subjA: *F* (1, 19) = 31.5, *p* < .0001, *η*_*p*_^2^ = .62; faceA: *F* (1, 9) = 18, *p* = .002, *η*_*p*_^2^ = .67). The three ways interaction was not reliable. As task interacted with expression or configuration, for brevity We unpacked the 2-ways interactions by computing a separate analysis for each task.

#### Experiment 3A: Eyes as the target

Replicating the results of experiment 1 and 2, participants were biased to indicate the presence of eyes in the context of fearful expression (main effect of expression: subjA: *F* (1, 19) = 37.1, *p* < .0001, *η*_*p*_^2^ = .66; faceA: *F* (1, 9) = 20.2, *p* = .002, *η*_*p*_^2^ = .69). The feature configuration also affected the ability to detect eyes (subjA: *F* (1, 19) = 64.77, *p* < .0001, *η*_*p*_^2^ = .77; faceA: *F* (1, 9) = 22.6, *p* = .001, *η*_*p*_^2^ = .72), showing larger bias to detect eyes in the context of a canonical face than a non-canonical face. Expression and configuration reliably interacted only for the subject-based analysis (subjA: *F* (1, 19) = 4.74, *p* = .04, *η*_*p*_^2^ = .20; faceA: *F* (1, 9) = 1, *p* = .34, *η*_*p*_^2^ = .10), though both analysis numerically showed the same pattern, with larger and more reliable configuration effect for happy (subjA: *t* (19) = 10.19, *p* < .0001; faceA: *t* (9) = 6.9, *p* < .0001) than for the fearful expressions (subjA: *t* (19) = 4.35, *p* < .0001; faceA: *t* (9) = 2.8, *p* < .018).

#### Experiment 3B: Mouth as the target

Happy mouth was detected better than fearful mouth, but the effect was only marginally reliable in the subject level analysis (subjA: *F* (1, 19) = 4.34, *p* = .051, *η*_*p*_^2^ = .19; faceA: *F* (1, 9) = .98, *p* = .38, *η*_*p*_^2^ = .099). There was a main effect of configuration (subjA: *F* (1, 19) = 5.9, *p* = .026, *η*_*p*_^2^ = .24; faceA: *F* (1, 9) = 7.37, *p* = .024, *η*_*p*_^2^ = .45), but unlike responding to eyes, participants were biased to indicate a presence of a mouth when it was presented in the scrambled (at the top) than in the canonical configuration (at the bottom). Expression and configuration did not interact (all *p*s > .5).

In summary, participants showed a stronger bias to identify fearful than happy features with the bias was stronger for fearful than happy eyes. The reverse pattern was observed for the mouth feature with a stronger bias to identify happy than fearful mouth. The impact of the feature configuration was also reversed depending on the target features. Participants showed a stronger bias to report the eyes in the canonical face configuration, whereas the tendency to recognise the mouth was stronger in the scrambled face configuration. We note that in both these cases, the target feature was presented at the top of the screen. Hence, it could be that participants overall showed more liberal response criterion for target at the upper part of an image.

### Effects across experiments

In these final analyses, we pooled the data from all the three experiments for two aims: 1) to formally evaluate the similarities and conflicting pattern of the results and 2) to assess whether knowledge about the stimuli statistics can predict the discriminability index, a hypothesis derived from the visual based account to the saliency of expressive features.

### Perceiving fearful eyes across experiments

We tested the robustness of the effects and their reproducibility using a 2(expression: fear and no fear) by 2(configuration: canonical and scrambled) as within-subject factors and the experiment as a between-subjects factor. For these analyses, we only used data that are comparable across the experiments. From experiment 1, we included the canonical condition and the scrambled condition where the eyes were presented at the bottom. We used all four conditions in experiment 2, and the four conditions from the eye detection task in experiment 3.

#### Discriminability index (d’) analysis

There was a main effect of experiment: (subjA: *F* (2, 52) = 9, *p* < .0001, *η*_*p*_^2^ = .83; faceA: *F* (2, 25) = 23.32, *p* < .0001, *η*_*p*_^2^ = .64) showing that overall discriminability of eyes was higher in experiment 3 than experiment 2 (subjA: *t* (30.8) = 3.03, *p* = .005; faceA: *t* (17.8) = 2.09, *p* = .051) and experiment 1 (subjA: *t* (36.4) = 3.98, *p* < .0001; faceA: *t* (8.23) = 12.6, *p* < .0001). The lower d’ of experiment 1 compared with experiment 2, was only reliable at the item-based analysis level (subjA: *t* (32.9) = 1.1, *p* = .29; faceA: *t* (12.1) = 5.1, *p* < .0001). Note that the statistical parameters reported here assumed unequal variances across the three experiments. This suggests that the filter had an impact, in which discriminating eyes from distorted eyes was easier when the distortion was done using a cubism filters that affected all spatial frequency than with a mosaic filter which affected the LSF primarily.

Across the three studies, we observed a strong effect of expression (subjA: *F* (1, 52) = 33.9, *p* < .0001, *η*_*p*_^2^ = .39; faceA: *F* (1, 25) = 100.86, *p* < .0001, *η*_*p*_^2^ = .80), showing higher d’ for fearful than non-fearful eyes. This effect did not interact with the experiments or the configuration of the features (all *p*s > .23). The effect of configuration did not reach significance, though there was a trend for an interaction between configuration and the experiments (subjA: *F* (2, 52) = 2.76, *p* = .072, *η*_*p*_^2^ = .09; faceA: *F* (2, 25) = 2.87, *p* = .075, *η*_*p*_^2^ = .19). This reflected the inconsistent pattern reported above for each individual experiment. Detecting eyes was superior in the scrambled than the canonical configuration in experiment 1. The reverse pattern was observed in experiments 2 and 3, though only three showed reliable configuration effect.

#### Criterion bias (c) analysis

Participants in the three experiments did not differ in the criterion biased they used when reporting eyes (all *p*s > .7). Across the three studies, we observed a strong effect of expression (subjA: *F* (1, 52) = 40.8, *p* < .0001, *η*_*p*_^2^ = .44; faceA: *F* (1, 25) = 36.1, *p* < .0001, *η*_*p*_^2^ = .59), showing stronger bias to report eyes in the context of fearful than non-fear expressions. This effect also interacted with the experiment, though was only reliable at the subject-based analysis (subjA: *F* (2, 52) = 4.14, *p* = .022, *η*_*p*_^2^ = .14; faceA: *F* (2, 25) = 2.44, *p* = .10, *η*_*p*_^2^ = .16). Across all three experiments, participants were more likely to report eyes in the canonical than the non-canonical faces (subjA: *F* (1, 52) = 54.59, *p* < .0001, *η*_*p*_^2^ = .51; faceA: *F* (1, 25) = 32.2, *p* < .0001, *η*_*p*_^2^ = .56). This canonical configuration bias was stronger for fearful than non-fearful expressions (expression by configuration interaction—subjA: *F* (1, 52) = 10.6, *p* = .002, *η*_*p*_^2^ = .14; faceA: *F* (1, 25) = 32.2, *p* < .0001, *η*_*p*_^2^ = .56). The experiments did not interact with the configuration manipulation.

#### Can power in low spatial frequency predict expressive feature saliency?

We first carried out a statistical analysis of the stimuli properties, namely the rectangles of the eyes and the mouth for each expression. For each stimulus, we computed the power in LSF following procedures reported in Rotshtein et al. [60]. Each feature stimulus was first normalized and was then transformed into the frequency domain using Fast Fourier Transform algorithm, implemented in Matlab. A Butterworth filter was used to keep frequencies below 8 cycles per image (viewed approximately as less than .625 cycles per degree). An average of the low frequencies power across the target feature rectangle was used in a repeated measure ANOVA to assess differences of expression (happy, neutral, and fear) and region of interest (ROI; eyes and mouth). Note, that neutral mouth was never a target in the current experiments, and that their values were only used here to make a full factorial design. This analysis included only the 8 faces that repeated across the three experiments. Given the hypothesis that expressive features would differ in their LSF power, we computed a set of *a priori t*-tests to assess differences between eyes and mouth as dependent on the expressions.

**Fig 5:**
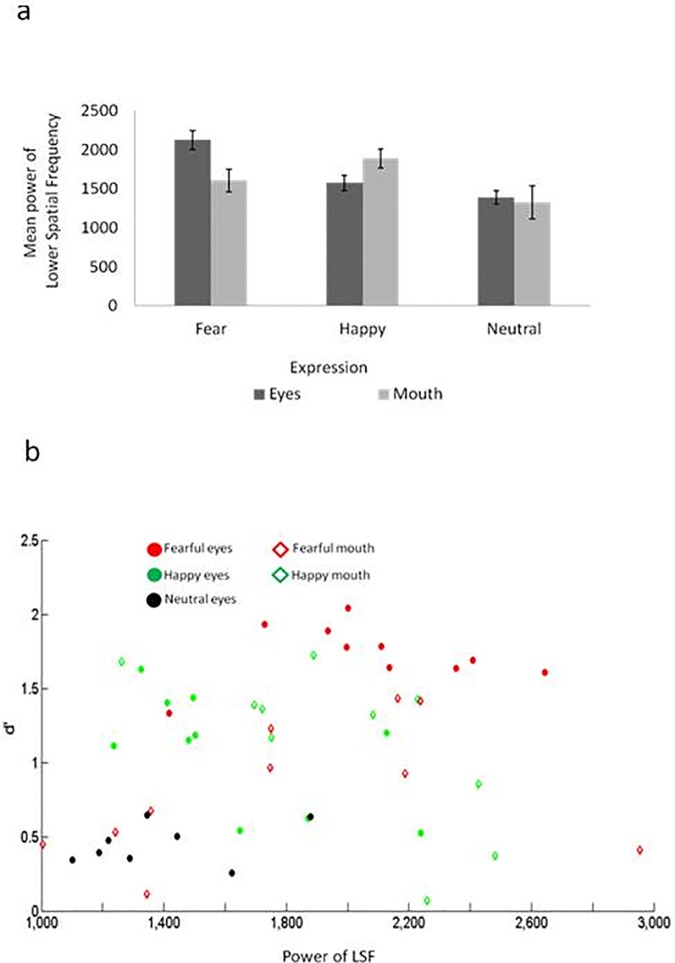
LSF power analysis. A. The charts represent the LSF power for each expressive feature. B. The scatterplots the LSF power against d’ for each target feature.

This analysis revealed a main effect of expression (*F* (2, 14) = 5.31, *p* = .02, *η*_*p*_^2^ = .56). A follow-up analysis collapsing across the mouth and eye region, showed that neutral features had overall less LSF power than fear (*t* (7) = 2.98, *p* = .02) and happy (*t* (7) = 2.54, *p* = .038). There was no difference in LSF power between happy and fearful features (*p* = .30). The interaction between ROI and expression approached significance (*F* (2, 14) = 3.9, *p* = .067, *η*_*p*_^2^ = .32). As the comparison between the expressional features was our a-priori focus of the current study, we followed the interaction by looking at its breakdown. This showed reliable differences in the power of the eyes between expressions (*F* (2, 14) = 13.3, *p* = .001, *η*_*p*_^2^ = .65) with fearful eyes showing larger LSF power than happy eyes (*t* (9) = 2.77, *p* = .022) and happy eyes showing larger LSF power than neutral eyes (*t* (7) = 2.89, *p* = .023). There were no reliable differences between expressions in the LSF power when considering the mouth rectangle, though there was a numerical difference (Fig 5A).

Finally, we wanted to test whether the LSF power of a target feature predicted the ability to discriminate this feature from noise. *d*’ was computed for each target feature (e.g., eyes and mouth) and each face, where we averaged results across the configuration conditions and participants, and the experiments when possible. Note that for the mouth features, *d’* was only based on the results of experiment 3, while *d’* for the neutral features were only based on the results of experiment 1. We had 48 data points (10 (faces) * 2 (expressions: fear, happy) * 2(targets: eyes, mouth) + 8 neutral eyes).

The relation between physical properties and perception follows Fechner law (e.g. [61]), which suggests that there is a logarithmic relation between perception and physical property of a stimulus. Indeed, a reliable linear relation where observed between item based d’ and the log10 of LSF power (Pearson *r* = .293, *p* = .043), which only approached linearity when using the absolute LSF power values (Pearson *r* = .245, *p* = .093). We note, that there were also reliable relations of item-based d’ and the power of LSF of the target feature when using non-parametric test, ensuring that the results were not driven by outliers (Spearman’s rho = .29, *p* = .044). For descriptive purpose, we scatter the power of the LSF against the d’ (Fig 5B).

## General discussion

The study aimed to test predictions derived from the visual-based hypothesis [6], proposed as an explanation for the perceptual saliency of diagnostic expressive features (e.g. eyes and mouth). We used signal detection theory to dissociate discriminability index, assumed to be more related to bottom-up sensory saliency, and criterion biased, assumed to reflect top-down processes. In line with the visual-based hypothesis, the results showed that fearful eyes and happy mouth are more accurately discriminable from noise than less expressive diagnostic features (e.g. neutral eyes, happy eyes, fearful mouth). The effect, however, was less reliable for the mouth region. Eyes had higher discriminability index than mouth. We, also, showed that this effect was independent of the features’ configuration, suggesting that, irrespective of whether a feature was presented in a canonical or scrambled face, it had no consistent impact on its detectability across the three experiments. An analysis of the low spatial frequency (LSF) power of the target features provided further support for the visual-based hypothesis. This analysis revealed that the expressive features had higher LSF power compared to neutral features, an effect that was primarily driven from differences between the eyes region. More interestingly, the power of LSF predicted the discriminability of the target features. Finally, the response bias was highly affected by the feature configuration and, also, by the expression. Participants were more likely to report that eyes were present in fearful expressions and canonical configuration. This suggests that the impact of affective content on perception is mediated via top down processes. We next discuss each of these effects in more details.

### Expressive feature effects on d’

The observation that detection of eyes was overall more precise than the detection of the mouth, irrespective of expression and configuration is consistent with prior literature (e.g., [5, 31, 48]). These results appear to contradict findings by Calvo and Nummenmaa [6, 62]. Using eye tracking, the authors concludes that information from the mouth is more salient than the eyes; as the first saccade was often directed to the mouth. However, there is an important difference between the task used in the current study and the tasks used by Calvo and Nummenmaa [6, 62]. The latter studies always used a task in which the facial expression was a relevant feature. For example, in the search task [6], participants had to identify the expressive face among neutral faces. This increased the relevance of the diagnostic expressive features through task demands. Therefore, it is not surprising that a diagnostic feature, like the mouth, would be salient. Another possibility is that the low-level visual properties of the expressive eyes and mouth used by Calvo and Nummenmaa [6] differed from the ones used in the current experiments. We used the Ekman and Friesen set [54], while Calvo and Nummenmaa [6, 62] used the Karolinska KDEF set. The Ekman and Friesen [54] are based on artificially posed expressions, which were aimed to exemplify the prototypical muscle movement associated with each expression as hypothesised by the authors; while the KDEF set used spontaneous expressions posed by actors. It could be that the eyes are deliberately prominent in the Ekman and Friesen’s set relative to the KDEF. Previous research has shown that participants used the most diagnostic information to complete any task, which depends on all the available stimuli set for a given experiment (targets and distracters; [33, 37]).

Importantly, the advantage of eyes was enhanced by fearful expressions. Fearful eyes were detected better than happy and neutral eyes. This is in line with previous literature, highlighting the important role of the eyes in processing fearful expression [2–4] (Experiment 6 in [6]) and [13, 22, 31, 36]. This study extends previous research to demonstrate that fearful eyes are perceptually salient even if expression is irrelevant to the task, and even after we have removed effects related to response bias. The analysis of LSF of the stimuli, mirrored to some degree the behavioural results, showing that fearful eyes had the highest LSF power.

In contrast to fearful eyes, smile was shown to be better detected than a fearful mouth. This observation is consistent with the idea that smiling mouth (relative to other expressive mouths) is perceptually salient based on bottom-up input [6, 62]. However, the effect of the smiling mouth was less reliable. While smiling mouth had numerically higher power in the LSF than fearful mouth, the difference was not reliable. This weak smiling effect may again be due to the specific properties of the stimuli set used, as the effect was reliable at the subject-based analysis but did not generalize across the face set. We note that most of the fearful expressions in the Ekman and Friesen’s set [54] bare their teeth in the fearful expressions, while not all the faces expressing happy smiles expose their teeth. Although we suggest that this effect is primarily driven by low level visual property of the target features, our findings, in line with the visual-based account, are likely to heavily depend on these properties and how a specific expression affect these in each face.

### Potential neural mechanisms mediating the expressive feature detection

The current study showed that the detection accuracy of expressive features is a product of their physical properties, e.g. a bottom up process. While this study was a pure behavioural study, it is tempting to speculate how it relates to knowledge gained in neuroscience. Specifically, we ask whether the findings reported here contradict previous observation showing automatic-like neural processing of emotional stimuli? Sensory processing of emotional laden stimulus is enhanced already at the early stages of the processing stream (e.g. [63, 64]). Here, we showed that the expressive features, had stronger LSF power than the non-expressive features (see previous section on LSF prediction of expressive feature saliency), hence they were likely to elicit larger response in early stages of sensory processing, as activation in these regions echoes the physical properties of the stimulus [65]. The impact of LSF is further magnified for briefly presented stimuli. It has been shown that briefly presented (~30ms) visual scene is primarily driven by input from the magnocellular pathways, which convey LSF information [66]. In support of the visual-based account, it is specifically shown that, via electroencephalographic oscillations (EEG), early neural to emotional and non-emotional scenes is differentiated based on the input conveyed by magnocellular pathways [67]. This is also in line with our observation that LSF power predicted the precision of detecting the target features. Taken together, our findings support the hypothesis that low-level visual properties drive the enhancement of sensory processing to emotional stimuli, making them salient based on their bottom up properties.

We also note that input from the magnocellular pathway arrives to the cortex before the parvocellular input arrives. It is hypothesised that magnocellular input provides a gist of the environment (stimulus) used to guide the following processing [40–42]. Hence, it is possible that the increase of LSF power of fearful eyes drives the saccades and attention processes to the eye regions of fearful faces. It is worth noting that the filter used here to compute LSF power maintain the known range of frequencies conveyed by the magnocellular pathway [68]. Although LSF is often taken as a marker for holistic processing, it is important to note that coarse processing can occur at multiple spatial scales: at the level of the face, but also at the level of the eyes. The coarse representations of eyes, typically shows two horizontal dark circles surrounded by brighter area, omitting details of the pupil, eye lashes, wrinkle, etc.

On the other hand, responses in sensory regions to emotional stimuli is shown to be mediated by the amygdala [37, 59, 63]. A critical question is whether the responses of the amygdala are driven by the sensory input irrespective of its affective value. The amygdala receives visual input from all the visual processing stages: from the early processing of the subcortical, pulvinar nuclei to the late cortical processing of the ventral and dorsal routes [69]. Consequently, the amygdala responds to non-emotional sensory input [70]. This suggests that the activity of the amygdala does not only reflect the affective/motivational value of the stimulus, but also the physical properties of the stimulus (as relayed by early sensory processing). However, it is important to note that amygdala responses to a ‘neutral’ stimulus is enhanced if it is learned to be associated with an affective value (through conditioning [63, 71]). Therefore, it could be argued that with experience, an association between low-level visual properties of the expressive features (LSF of fearful eyes) and an emotional value (fear) is formed. This may lead to an increase in amygdala responses to these features which is then projected back to the early sensory cortices.

### Effect of expression and configuration on response bias but not d’

The configuration of the features had no consistent impact on the ability to detect the expressive features. This finding is in line with previous studies showing that performances on search task were minimally affected by the configuration of the target features [6]. This suggests that the facial configuration does not affect the precision detection of a single facial feature. In other words, faces can be represented at the level of individual features. There is evidence that suggests that features are processed independently at least at the initial stages of processing [64]. This may appear to contradict a large amount of literature arguing for holistic face processing [72], specifically for facial expressions [30]. It should be noted that in the current study, the face was presented as three separate featural-stripes (Figs 1A and 3A). This means that even in the canonical condition, the stimuli did not look like a complete face. Therefore, the stimuli configuration used here may have led participants to adopt a feature-based over a holistic analysis.

We observed that configuration reliably affected the response bias. Participants were more likely to indicate that the eyes were present in the context of a canonical than non-canonical configuration–leading to an increase in hits and false alarms. The observation that the face configuration affected response bias has been reported before [73, 74]. In fact, it has been argued that studies of holistic face processing who only report accuracy, often confound response bias and decision precision leading to inaccurate interpretations of the results (see review by [75]); erroneously attributing increase accuracy to better detection of a feature.

The robust effect of configuration on response bias but not d’, observed here, is in line with the attentional-based hypothesis for holistic face perception [76]. The attentional-based hypothesis, suggest that the parts of the face are encoded separately, but a strategic allocation of attention, guided by experience, ‘enforces’ the grouping of these features. Based on extensive experience, this grouping process become the default mechanism for processing the individual face parts. It gives rise to automatic-like processing of face as a single unit. The attentional-based hypothesis is an alternative to the face-template hypothesis which argues that a face is a single unit of processing (e.g., [77]). We suggest that, in this experiment, configuration effects on feature detection were the result of a top-down strategic decision, based on strategic grouping of the features.

Why would canonical configuration be associated with more liberal criterion of detection eyes? It could be that the activation of the entire face led to a filling-in phenomenon leading to an increased number of false alarms. Visual filling-in has been shown to be stronger when items are more likely to be grouped together [78], as the case of faces.

Surprisingly, a bias to detect mouth was larger in the scrambled than the canonical condition. When mouth was presented at the top, participants were more likely to report its presence. Experiment 1 showed similar results, a stronger response bias for top followed by middle and bottom targets in decreasing order. This raises an interesting possibility that response biases and detection threshold are set differently across the visual field with more liberal detection thresholds for targets located higher than lower in the visual field.

Furthermore, response bias was affected by the emotions. Participant were more likely to report the presence of an eye in the context of fearful than non-fearful expressions, which may have been partially driven by the fearful mouth. It could be that the fearful mouth led to an overall increase signalling of fear, biasing the decision toward a more liberal detection threshold. Change in response bias (primarily driven by increase in false alarms) to emotional stimuli is a common phenomenon and often reported for emotional memories (e.g., [79]). It should, also, be noted that it is possible that our filtering procedure (used to create the no-eye feature) may have led to an increase in false alarms. As the no-eyes feature maintained some residuals properties of eyes, which under limited presentation condition may have been misinterpreted by the visual system, which may lead to an increase in false alarms.

### Methodological consideration

In the light of the recent reproducibility crisis [80], the current study opted for partial internal replication approach. Here we conducted three experiments, each recruiting a moderate sample of participants (n = ~15), in which the three experiments tested the same theoretical question, but along different parameters. The aim was to provide a conceptual replication. We, also, provided an analysis that collapsed across all participants, to demonstrate the robustness of the effect in a large sample. We note that as the sample of each experiment was moderate and the designs were also slightly different, results did not always concur. This was specifically the case when considering the effect of facial configuration, and the interaction of expression and configuration on the *d*’ measure. Here, we assumed that if observations were not replicated across the three experiments, it means they are not statistical robust and hence should be assumed to be false positives when they do occur.

In addition, to further assess the robustness of the observed effects and provide additional analysis using a different parameter space [81]. We computed two separate analyses for each experiment. In one analysis, we treated the participants as a random variable and in a second we treated the stimuli as the random variable. This ensure that the results can be generated beyond the specific samples of the participants or the faces.

## Conclusions

In this research, we investigated the perceptual saliency of the eyes and mouth as a function of facial expression and configuration via signal detection theory. The present results showed that the eyes are detected better than the mouth. Fearful eyes were detected better than no-fearful eyes (happy or neutral); while smiling mouth was detected better than fearful mouth. These effects were independent of the facial feature configuration indicating that the expressive features themselves facilitated perception. Mirroring the behaviour, we also showed that expressive features have stronger power of LSF, an effect that was driven by the eyes. Importantly, the power of LSF predicted the ability to detect the features. Finally, response bias was affected by the configuration of the features and their expression with a higher bias to report eyes in the context of canonical and fearful faces. The results support the visual-based hypotheses suggesting low-level visual properties drive the perceptual saliency of expressive features. The data, also, showed that emotion and configuration contribute to prioritization through top-down processes.

## Supporting information

S1 Fig Results for Experiment 1. The overall data pertaining to experiment 1.
(XLSX)

S2 Fig Results for Experiment 2. The overall data pertaining to experiment 2.
(XLSX)

S3 Fig Results for Experiment 3a. The overall data pertaining to experiment 3a.
(XLSX)

S4 Fig Results for Experiment 3b. The overall data pertaining to experiment 3b.
(XLSX)

## References

[1] Kohler C. G., Turner T., Stolar N. M., Bilker W. B., Brensinger C. M., Gur R. E. et al (2004). Differences in facial expressions of four universal emotions. Psychiatry research, 128(3), 235–244. 10.1016/j.psychres.2004.07.003 15541780

[2] Smith M. L., Cottrell G. W., Gosselin F., & Schyns P. G. (2005). Transmitting and decoding facial expressions. Psychological Science, 16(3), 184–189. 10.1111/j.0956-7976.2005.00801.x 15733197

[3] Wallace S., Coleman M., & Bailey A. (2008). An investigation of basic facial expression recognition in autism spectrum disorders, 22(7), 37–41.

[4] Adolphs R., Gosselin F., Buchanan T. W., & Tranel D. (2005). A mechanism for impaired fear recognition after amygdala damage, Nature, 433(7021), 68–72. 10.1038/nature03086 15635411

[5] Djukic A., Rose S. A., Jankowski J. J., & Feldman J. F. (2014). Rett Syndrome: Recognition of Facial Expression and Its Relation to Scanning Patterns. Pediatric Neurology, 51(5), 650–656. 25217338 10.1016/j.pediatrneurol.2014.07.022

[6] Calvo M. G., & Nummenmaa L. (2008). Detection of emotional faces: salient physical features guide effective visual search. Journal of Experimental Psychology: General, 137(3), 471. 18729711 10.1037/a0012771

[7] Öhman A. (1993). Fear and anxiety as emotional phenomena: Clinical phenomenology, evolutionary perspectives, and information processing mechanisms *In* Lewis M. & Haviland J. M. (Eds.), Handbook of emotions (pp. 511–536). New York: Guilford Press

[8] Öhman A., & Mineka S. (2001). Fears, phobias, and preparedness: toward an evolved module of fear and fear learning. Psychological review, 108(3), 483 11488376 10.1037/0033-295x.108.3.483

[9] Kraut R. E., & Johnston R. E. (1979). Social and emotional messages of smiling: An ethological approach. Journal of personality and social psychology, 37(9), 1539.

[10] Parkhurst D., Law K., & Niebur E. (2002). Modelling the role of salience in the allocation of overt visual attention. Vision research, 42(1), 107–123. 11804636 10.1016/s0042-6989(01)00250-4

[11] Kaplan E., & Shapley R.M. (1986). The primate retina contains two types of ganglion cells, with high and low contrast sensitivity. Proceedings of National Academy of Sciences USA, 83, 2755–2757. 10.1073/pnas.83.8.2755 PMC323379 3458235

[12] Avidan G., Harel M., Hendler T., Ben-Bashat D., Zohary E., & Malach R. (2002). Contrast sensitivity in human visual areas and its relationship to object recognition. Journal of Neurophysiology, 87(6), 3102–3116. 12037211 10.1152/jn.2002.87.6.3102

[13] Whalen P. J., Kagan J., Cook R. G., Davis F. C., Kim H., Polis S et al (2004). Human amygdala responsivity to masked fearful eye whites. Science, 306(1), 2061. 15604401 10.1126/science.1103617

[14] Kobayashi H., & Kohshima S. (1997). Unique morphology of the human eye. Nature, 387(1), 766–767. 10.1038/42842 9194557

[15] Vuilleumier P. (2005). How brains beware: neural mechanisms of emotional attention. Trends in Cognitive Sciences, 9(12), 585–94. 10.1016/j.tics.2005.10.011 16289871

[16] Dolan R. J., & Vuilleumier P. (2003). Amygdala automaticity in emotional processing. Annual New York Academy of Sciences, 1, 348–355. 10.1111/j.1749-6632.2003.tb07093.x 12724170

[17] Peossa L., Oilveira L., & Pereira M. (2013). Top-down attention and processing of emotional stimuli. The Cambridge Handbook of Affective Neuroscience, 357–374.

[18] Treue S. (2003). Visual attention: the where, what, how and why of saliency. Current opinion in neurobiology, 13(4), 428–432. 12965289 10.1016/s0959-4388(03)00105-3

[19] Janik S. W., Wellens R., Goldberg M. L., & Dell’Osso L. F. (1978). Eyes as the center of focus in the visual examination of human faces. Perceptual and Motor Skills, 47, 857–858. 10.2466/pms.1978.47.3.857 740480

[20] Lewkowicz D. J., & Hansen-Tift A. M. (2012). Infants deploy selective attention to the mouth of a talking face when learning speech. Proceedings of the National Academy of Sciences, 109(5), 1431–1436. 10.1073/pnas.1114783109 PMC3277111 22307596

[21] Gulberg M. (2003). Eye movements and gestures in human face-to-face interaction *In* Hyona J., Radach R., & Deubel H. (Eds.), The mind's eyes: Cognitive and applied aspects of eye movements (pp. 695–703). Oxford: Elsevier.

[22] Spezio M. L., Huang P.-Y. S., Castelli F., & Adolphs R. (2007). Amygdala damage impairs eye contact during conversations with real people. The Journal of Neuroscience: The Official Journal of the Society for Neuroscience, 27(15), 3994–3997. 17428974 10.1523/JNEUROSCI.3789-06.2007 PMC6672539

[23] Jones W., & Klin A. (2013). Attention to eyes is present but in decline in 2-6-month-old infants later diagnosed with autism. Nature, 504(7480), 427–431. 10.1038/nature12715 24196715 PMC4035120

[24] Keating C. F., & Keating E. G. (1982). Visual scan patterns of rhesus monkeys viewing faces. Perception, 11(2), 211–219. 10.1068/p110211 7155774

[25] Emery N. J., Lorincz E. N., Perrett D. I., Oram M. W., & Baker C. I. (1997). Gaze following and joint attention in rhesus monkeys (Macaca mulatta). Journal of comparative psychology, 111(3), 286 9286096 10.1037/0735-7036.111.3.286

[26] Kaplan G., & Rogers L. J. (2002). Patterns of gazing in orangutans (Pongo pygmaeus). International Journal of Primatology, 23(3), 501–526.

[27] Emery N. J. (2000). The eyes have it: the neuroethology, function and evolution of social gaze. Neuroscience & Biobehavioral Reviews, 24(6), 581–604. 10940436 10.1016/s0149-7634(00)00025-7

[28] Ekman P., & Friesen W.V. (1975a). Unmasking the face. Englewood Cliffs, NJ: Prentice Hall.

[29] Gosselin F., & Schyns P. G. (2001). Bubbles: a technique to reveal the use of information in recognition tasks. Vision research, 41(17), 2261–2271. 11448718 10.1016/s0042-6989(01)00097-9

[30] Calder A. J., Young A. W., Keane J., & Dean M. (2000). Configural information in facial expression perception. Journal of Experimental Psychology: Human perception and performance, 26(2), 527 10811161 10.1037//0096-1523.26.2.527

[31] Eisenbarth H., & Alpers G. W. (2011). Happy mouth and sad eyes: Scanning emotional facial expressions. Emotion, 11(4), 860–865. 10.1037/a0022758 21859204

[32] Gillespie S. M., Rotshtein P., Wells L. J., Beech A. R., & Mitchell I. J. (2015). Psychopathic traits are associated with reduced attention to the eyes of emotional faces among adult male non-offenders. Frontiers in human neuroscience, 9. 10.3389/fnhum.2015.00552 PMC4595655 26500524

[33] Schurgin M. W., Nelson J., Iida S., Ohira H., Chiao J. Y., & Franconeri S. L. (2014). Eye movements during emotion recognition in faces. Journal of vision, 14(13), 14–14. 10.1167/14.13.14 25406159

[34] Wells L. J., Gillespie S. M., & Rotshtein P. (2016). Identification of Emotional Facial Expressions: Effects of Expression, Intensity, and Sex on Eye Gaze. PloS one, 11(12), e0168307 10.1371/journal.pone.0168307 27942030 PMC5152920

[35] Gamer M., & Buchel C. (2009). Amygdala activation predicts gaze towards fearful eyes. The Journal of Neuroscience, 29 (28), 9123–9126. 10.1523/JNEUROSCI.1883-09.2009 19605649 PMC6665435

[36] Morris J. S., DeBonis M., & Dolan R. J. (2002). Human amygdala responses to fearful eyes. Neuroimage, 17(1), 214–222. 12482078 10.1006/nimg.2002.1220

[37] Rotshtein P., Richardson M. P., Winston J. S., Kiebel S. J., Vuilleumier P., Eimer M. et al (2010). Amygdala damage affects event‐related potentials for fearful faces at specific time windows. Human brain mapping, 31(7), 1089–1105. 10.1002/hbm.20921 20017134 PMC3173845

[38] Vuilleumier P., Armony J.L., Driver J., & Dolan R.J. (2003). Distinct spatial frequency sensitivities for processing faces and emotional expressions. Nature Neuroscience, 6, 624–631 10.1038/nn1057 12740580

[39] Winston J.S., Vuilleumier P., & Dolan R. J. (2003). Effects of low-spatial frequency components of fearful faces on fusiform cortex activity. Current Biology, 13, 1824–1829. 14561410 10.1016/j.cub.2003.09.038

[40] Bullier J. (2001). Integrated model of visual processing. Brain Research Reviews, 36(2), 96–107. 11690606 10.1016/s0165-0173(01)00085-6

[41] Hegdé J. (2008). Time course of visual perception: coarse-to-fine processing and beyond. Progress in neurobiology, 84(4), 405–439. 10.1016/j.pneurobio.2007.09.001 17976895

[42] Kveraga K., Boshyan J., & Bar M. (2007). Magnocellular projections as the trigger of top-down facilitation in recognition. The Journal of Neuroscience, 27(48), 13232–13240. 10.1523/JNEUROSCI.3481-07.2007 18045917 PMC6673387

[43] Calvo M. G., Fernández-Martín A., & Nummenmaa L. (2012). Perceptual, categorical, and affective processing of ambiguous smiling facial expressions. Cognition, 125, 273–293. 10.1016/j.cognition.2012.07.021 22939734

[44] Calvo M. G., & Beltrán D. (2013). Recognition advantage of happy faces: tracing the neurocognitive processes. Neuropsychologia, 51(11), 2051–2061. 10.1016/j.neuropsychologia.2013.07.010 23880097

[45] daSilva E.B., Cragar K., Geisler D., Newbern P., Orem B., & Puce A. (2016). Something to sink your teeth into: The presence of teeth augments ERPs to mouth expressions. NeuroImage, 127, 227–241. 10.1016/j.neuroimage.2015.12.020 26706446

[46] Adolphs R. (2002). Recognizing emotion from facial expressions: psychological and neurological mechanisms. Behavioral and cognitive neuroscience reviews, 1(1), 21–62. 17715585 10.1177/1534582302001001003

[47] Nummenmaa L., & Calvo M. G. (2015). Dissociation between recognition and detection advantage for facial expressions: A meta-analysis. Emotion, 15(2), 243 10.1037/emo0000042 25706834

[48] Le S., Raufaste E., & Demonet J. F. (2003). Processing of normal, inverted, and scrambled faces in a patient with prosopagnosia: behavioural and eye tracking data. Cognitive Brain Research, 17(1), 26–35. 12763189 10.1016/s0926-6410(03)00077-6

[49] McKelvie S. J. (1995). Emotional expression in upside‐down faces: Evidence for configurational and componential processing. British Journal of Social Psychology, 34(3), 325–334. 7551775 10.1111/j.2044-8309.1995.tb01067.x

[50] Stanislaw H., & Todorov N. (1999). Calculation of signal detection theory measures. Behavior research methods, instruments, & computers, 31(1), 137–149. 10.3758/bf03207704 10495845

[51] Stone A., & Valentine T. (2004). Better the devil you know? Nonconscious processing of identity and affect of famous faces. Psychonomic Bulletin & Review, 11(3), 469–474. 15376797 10.3758/bf03196597

[52] Kirouac G., & Doré F. Y. (1984). Judgment of facial expressions of emotion as a function of exposure time. Perceptual and Motor Skills, 59, 147–150. 10.2466/pms.1984.59.1.147 6493929

[53] Ogawa T., & Suzuki N. (1999). Response differentiation to facial expression of emotion as increasing exposure duration. Perceptual and Motor Skills, 89, 557–563. 10597592

[54] Ekman P., & Friesen W. V. (1975b). Pictures of facial affect. Consulting Psychologists Press.

[55] Kanai R., & Rees G. (2011). The structural basis of inter-individual differences in human behaviour and cognition. Nature Reviews Neuroscience, 12(4), 231–242. 10.1038/nrn3000 21407245

[56] Tanaka J. W., & Farah M. J. (1993). Parts and wholes in face recognition. T*he Quarterly journal of experimental psychology*, 46(2), 225–245. 8316637 10.1080/14640749308401045

[57] Spillmann L., & Werner J. S. (1996). Long-range interactions in visual perception. Trends in neurosciences, 19(10), 428–434. 8888520 10.1016/0166-2236(96)10038-2

[58] Wannig A., Stanisor L., & Roelfsema P. R. (2011). Automatic spread of attentional response modulation along Gestalt criteria in primary visual cortex. Nature neuroscience, 14(10), 1243–1244. 10.1038/nn.2910 21926984

[59] Vuilleumier P., Armony J. L., Driver J., & Dolan R. J. (2001). Effects of attention and emotion on face processing in the human brain: an event-related fMRI study. Neuron, 30(3), 829–841. 11430815 10.1016/s0896-6273(01)00328-2

[60] Rotshtein P., Schofield A., Funes M. J., & Humphreys G. W. (2010b). Effects of spatial frequency bands on perceptual decision: It is not the stimuli but the comparison. Journal of vision, 10(10), 25–25. 10.1167/10.10.25 20884490

[61] Stevens S. S. (1957). On the psychophysical law. Psychological review, 64(3), 153 13441853 10.1037/h0046162

[62] Calvo M.G. & Nummenmaa L. (2011). Time course of discrimination between emotional facial expressions: The role of visual saliency. Vision Research, 51, 1751–1759. 10.1016/j.visres.2011.06.001 21683730

[63] Quirk G. J., Armony J. L., & LeDoux J. E. (1997). Fear conditioning enhances different temporal components of tone-evoked spike trains in auditory cortex and lateral amygdala. Neuron, 19(3), 613–624. 9331352 10.1016/s0896-6273(00)80375-x

[64] Rotshtein P., Geng J. J., Driver J., & Dolan R. J. (2007). Role of features and second-order spatial relations in face discrimination, face recognition, and individual face skills: behavioral and functional magnetic resonance imaging data. Journal of Cognitive Neuroscience, 19(9), 1435–1452. 10.1162/jocn.2007.19.9.1435 17714006 PMC2600425

[65] Zhang X., Zhaoping L., Zhou T., & Fang F. (2012). Neural activities in V1 create a bottom-up saliency map. Neuron, 73(1), 183–192. 10.1016/j.neuron.2011.10.035 22243756

[66] Delorme A., Richard G., & Fabre-Thorpe M. (1999). Rapid processing of complex natural scenes: A role for the magnocellular visual pathways?. Neurocomputing, 26, 663–670.

[67] Miskovic V., Martinovic J., Wieser M. J., Petro N. M., Bradley M. M., & Keil A. (2015). Electrocortical amplification for emotionally arousing natural scenes: The contribution of luminance and chromatic visual channels. Biological psychology, 106, 11–17. 10.1016/j.biopsycho.2015.01.012 25640949 PMC4361366

[68] Skottun B. C. (2000). The magnocellular deficit theory of dyslexia: the evidence from contrast sensitivity. Vision research, 40(1), 111–127. 10768046 10.1016/s0042-6989(99)00170-4

[69] Pessoa L., & Adolphs R. (2010). Emotion processing and the amygdala: from a'low road' to 'many roads' of evaluating biological significance. Nature reviews neuroscience, 11(11), 773–783. 10.1038/nrn2920 20959860 PMC3025529

[70] Romanski L. M., Clugnet M. C., Bordi F., & LeDoux J. E. (1993). Somatosensory and auditory convergence in the lateral nucleus of the amygdala. Behavioral neuroscience, 107(3), 444 8329134 10.1037//0735-7044.107.3.444

[71] Uwano T., Nishijo H., Ono T., & Tamura R. (1995). Neuronal responsiveness to various sensory stimuli, and associative learning in the rat amygdala. Neuroscience, 68(2), 339–361. 7477945 10.1016/0306-4522(95)00125-3

[72] Maurer D., Le Grand R., & Mondloch C. J. (2002). The many faces of configural processing. Trends in cognitive sciences, 6(6), 255–260. 12039607 10.1016/s1364-6613(02)01903-4

[73] Richler J. J., Gauthier I., Wenger M. J., & Palmeri T. J. (2008). Holistic processing of faces: perceptual and decisional components. Journal of Experimental Psychology: Learning, Memory, and Cognition, 34(2), 328 10.1037/0278-7393.34.2.328 18315409

[74] Richler J. J., Cheung O. S., & Gauthier I. (2011). Beliefs alter holistic face processing… if response bias is not taken into account. Journal of Vision, 11(13), 17–17. 10.1167/11.13.17 22101018 PMC3354002

[75] Richler J. J., & Gauthier I. (2014). A meta-analysis and review of holistic face processing. Psychological Bulletin, 140(5), 1281 10.1037/a0037004 24956123 PMC4152424

[76] Richler J., Palmeri T. J., & Gauthier I. (2012). Meanings, mechanisms, and measures of holistic processing. Frontiers in Psychology, 3, 553 10.3389/fpsyg.2012.00553 23248611 PMC3520179

[77] Farah M. J., Wilson K. D., Drain M., & Tanaka J. N. (1998). What is" special" about face perception?. Psychological review, 105(3), 482 9697428 10.1037/0033-295x.105.3.482

[78] Zhaoping L., Jingling L. (2008). Filling-In and Suppression of Visual Perception from Context: A Bayesian Account of Perceptual Biases by Contextual Influences. PLOS Computational Biology, 4(2), e14 10.1371/journal.pcbi.0040014 18282080 PMC2242827

[79] Dougal S., & Rotello C. M. (2007). “Remembering” emotional words is based on response bias, not recollection. Psychonomic Bulletin & Review, 14(3), 423–429. 17874582 10.3758/bf03194083

[80] Open Science Collaboration. (2015). Estimating the reproducibility of psychological science. Science, 349(6251), aac4716 10.1126/science.aac4716 26315443

[81] Steegen S., Tuerlinckx F., Gelman A., & Vanpaemel W. (2016). Increasing transparency through a multiverse analysis. P*erspectives on Psychological Science*, 11(5), 702–712. 10.1177/1745691616658637 27694465

