# Correction: The perceptual saliency of fearful eyes and smiles: A signal detection study

**DOI:** 10.1371/journal.pone.0179149

**Published:** 2017-06-01

**Authors:** Mahmoud Medhat Elsherif, Muhammet Ikbal Sahan, Pia Rotshtein

The second author's name is spelled incorrectly. The correct name is: Muhammet Ikbal Sahan. The correct citation is: Elsherif MM, Sahan MI, Rotshtein P (2017) The perceptual saliency of fearful eyes and smiles: A signal detection study. PLoS ONE 12(3): e0173199. https://doi.org/10.1371/journal.pone.0173199
